# In situ analysis of osmolyte mechanisms of proteome thermal stabilization

**DOI:** 10.1038/s41589-024-01568-7

**Published:** 2024-02-29

**Authors:** Monika Pepelnjak, Britta Velten, Nicolas Näpflin, Tatjana von Rosen, Umberto Capasso Palmiero, Jeong Hoon Ko, Heather D. Maynard, Paolo Arosio, Eilika Weber-Ban, Natalie de Souza, Wolfgang Huber, Paola Picotti

**Affiliations:** 1 Department of Biology, Institute of Molecular Systems Biology, ETH Zurich, Zurich, Switzerland; 2 Division of Computational Genomics and Systems Genetics, German Cancer Research Center (DKFZ), Heidelberg, Germany; 3 Centre for Organismal Studies (COS) & Center for Scientific Computing (IWR), Heidelberg University, Heidelberg, Germany; 4 Department of Biology, Institute of Molecular Biology & Biophysics, ETH Zurich, Zurich, Switzerland; 5 Department of Chemistry and Applied Biosciences, Institute of Chemical and Bioengineering, ETH Zurich, Zurich, Switzerland; 6 Department of Chemistry and Biochemistry, University of California, Los Angeles, CA USA; 7 Department of Quantitative Biomedicine, University of Zurich, Zurich, Switzerland; 8 Genome Biology Unit, European Molecular Biological Laboratory, Heidelberg, Germany

**Keywords:** Protein aggregation, Proteomics, Mechanism of action, Mass spectrometry, Small molecules

## Abstract

Organisms use organic molecules called osmolytes to adapt to environmental conditions. In vitro studies indicate that osmolytes thermally stabilize proteins, but mechanisms are controversial, and systematic studies within the cellular milieu are lacking. We analyzed *Escherichia coli* and human protein thermal stabilization by osmolytes in situ and across the proteome. Using structural proteomics, we probed osmolyte effects on protein thermal stability, structure and aggregation, revealing common mechanisms but also osmolyte- and protein-specific effects. All tested osmolytes (trimethylamine *N*-oxide, betaine, glycerol, proline, trehalose and glucose) stabilized many proteins, predominantly via a preferential exclusion mechanism, and caused an upward shift in temperatures at which most proteins aggregated. Thermal profiling of the human proteome provided evidence for intrinsic disorder in situ but also identified potential structure in predicted disordered regions. Our analysis provides mechanistic insight into osmolyte function within a complex biological matrix and sheds light on the in situ prevalence of intrinsically disordered regions.

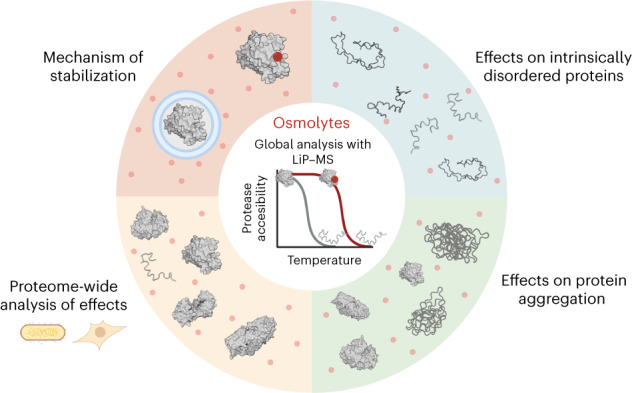

## Abstract (web summary)

A proteome-wide thermal profiling study of osmolyte action on *E. coli* and human proteins within the cellular milieu reveals mechanisms of protein thermal stabilization by osmolytes and in situ behavior of intrinsically disordered proteins.

## Main

Adaptation to acute environmental changes is crucial for organism survival. A key adaptation mechanism under stress conditions is the accumulation of osmolytes, small uncharged organic compounds that regulate osmotic pressure in cells^1–4^. In addition to their ability to control cell water content, osmolytes stabilize lipid membranes^5^ and protect proteins against denaturation^6–11^.

Much remains to be understood about osmolyte mechanisms of action. In vitro studies on monomeric, purified proteins have revealed that osmolytes stabilize proteins against thermal denaturation^1–3^, but the mechanisms of stabilization are controversial. Models of osmolyte-dependent thermal stabilization include the preferential exclusion model^8,12–16^, which implies that osmolytes form unfavorable interactions with the protein backbone and render the unfolded state energetically unfavorable. Other models propose effects mediated by an increase in medium viscosity^17^ or crowding^18^ or protein stabilization via direct osmolyte binding^19,20^. Some studies have reported that the native structure of a protein is not affected by osmolyte addition^21,22^, but native structure is not preserved for all osmolyte–protein combinations^23–25^. Osmolytes have been shown to both reduce^26–30^ and promote aggregation^31–36^ as well as change the structure of aggregates^37,38^.

Importantly, because most previous studies on osmolyte mechanisms were based on individual purified proteins^1–3,8,15,39^, they did not account for effects of the cellular matrix, which could profoundly affect protein thermal stability and osmolyte-dependent stabilization^40^. Also, results on a few individual proteins may not apply across the proteome. Systematic, proteome-wide studies of osmolyte mechanisms within the complex cellular matrix are needed.

We adapted a global structural proteomics method based on limited proteolysis and mass spectrometry (LiP–MS) to the study of osmolyte mechanisms^41–43^. Our approach enables the measurement of protein stability, structure and aggregation in a cellular lysate and on a proteome-wide scale^42^. We could therefore apply it to test the effects of all four major groups of osmolytes (sugars, polyols, methylamines and amino acids) on the proteome of *E. coli*, which uses osmolytes to counteract stress^44,45^, and to test their previously proposed mechanisms of action. All tested osmolytes stabilized many proteins; methylamine trimethylamine *N*-oxide (TMAO) stabilized the largest number of proteins and with the strongest effect, and glycerol stabilized the fewest proteins. Interestingly, the preferential exclusion model could explain much of the observed stabilization effect across the proteome. Our approach allows the assessment of whether direct osmolyte binding is required for protein stabilization and reveals strong stabilization of a few proteins due to protein–osmolyte binding events, but that binding is not broadly necessary for stabilization. Our global study enabled the analysis of the biophysical properties of osmolyte-stabilized proteins, showing preferential stabilization of proteins with lower charge and lower isoelectric point (pI) across osmolytes, whereas glucose and trehalose stabilized those rich in negatively charged residues. Osmolytes generally caused an upward shift in the temperatures at which proteins aggregated due to stabilization of protein structure. Similar effects could be seen for TMAO and trehalose in the human (HEK293T cell) proteome.

We studied the thermal behavior and the effects of osmolytes on intrinsically disordered proteins in the human proteome. We found evidence of intrinsic disorder (that is, flat thermal unfolding profiles) in situ, and more than one-third of protein regions predicted to be disordered may be folded in the cellular context. TMAO had different effects on folded and disordered human proteins, stabilizing globular proteins but promoting aggregation of large disordered ones.

We provide a systematic study of in situ osmolyte mechanisms across an entire proteome. The data revealed both general and protein-specific effects of osmolytes on protein stabilization and aggregation and identified biophysical principles by which these effects occur.

## Results

### LiP–MS for the study of proteome-wide osmolyte effects

**Fig. 1:**
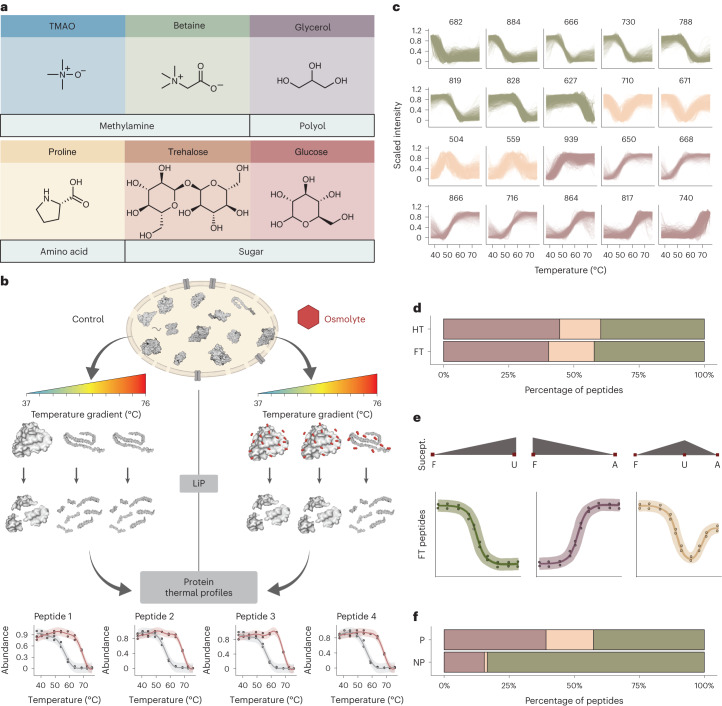
LiP–MS thermal profiling to study osmolyte effects. **a**, Chemical structures of osmolytes used in the study. All osmolytes were used at 1 M except for trehalose (0.5 M). **b**, Overview of the experimental procedure. Aliquots of an *E. coli* lysate were subjected to a thermal gradient (ten temperatures; 37–76 °C) in the presence or absence of an osmolyte. Subsequent proteolysis with PK under native conditions yielded proteolytic fragments that are informative about the folded state of proteins, with PK accessibility increasing after protein unfolding. Trypsin digestion under denaturing conditions generated peptides that can be measured with MS. Scaled relative abundance of individual peptides across the temperature gradient allows profiling of protein thermal unfolding, and osmolyte effects can be studied by comparing profiles between control (gray curves) and osmolyte (red curves) conditions. Figure created with BioRender.com. **c**, Peptide thermal profile clusters in the absence of osmolytes. Both fully tryptic (FT) and half-tryptic (HT) peptides are shown. The numbers of peptides in each cluster are indicated. Colors indicate profiles with decreasing intensity (group 1, green), profiles with increasing intensity (group 2, purple) and nonmonotonous profiles (group 3, orange). **d**, Percentage of cluster groups from **c** shown separately for FT and HT peptides. **e**, Interpretation of FT peptide behavior in the three cluster groups from **c**. We interpret changing FT peptide intensity as a function of increased or decreased proteolytic susceptibility (Suscept.) as indicating protein in a folded (F), unfolded (U) or aggregated (A) state. For HT peptides, the opposite effect (that is, a flipped thermal profile) is expected (Supplementary Fig. 1f). **f**, Percentage of cluster groups from **c** separated by proteins defined as nonprecipitators (NP) and precipitators (P) in TPP; only FT peptides are shown (see Supplementary Fig. 1g for HT peptides).

We previously showed that LiP–MS applied over a range of temperatures can measure protein thermostability across the proteome^42^. In brief, sequence-unspecific proteinase K (PK) is added to aliquots of a native lysate exposed to a temperature gradient and cleaves flexible and accessible regions of proteins. As proteins unfold with increasing temperature, PK increasingly cleaves newly accessible regions. The abundance of differentially produced peptides across the temperature gradient can then be monitored to yield thermal profiles for each protein. In our previous work, we used such profiles to extract protein melting temperatures at the proteome scale^42^. Here, we improved the coverage and resolution of the approach, extended it to enable robust comparison of thermal profiles between conditions (Fig. 1a,b) and made it compatible with the analysis of small-molecule osmolytes.

First, our new thermal profiling pipeline included analysis of both half-tryptic and fully tryptic peptides and not just the latter as in our previous work^42^ (Supplementary Fig. 1a and Methods). Half-tryptic peptides are a rich source of structural information, and the intensities of both peptide types are well correlated (Supplementary Fig. 1b). Including half-tryptic peptides thus increased the structural resolution and sequence coverage of the analysis.

Second, we extended the approach beyond the analysis of pure protein unfolding profiles (Methods) to capture nonmonotonous behavior and other types of complex structural changes induced by an increase in temperature, including protein aggregation. Third, we ensured that PK activities stayed constant under different conditions, for example, at different temperatures or after the addition of osmolytes. We found that temperature in the tested range (37–76 °C) and osmolytes at a concentration of 1 M had only small effects on PK activity (6.6% and 3.2% of peptides, respectively; Supplementary Fig. 1c,d and Methods), which could be further reduced by scaling peptide intensities (Supplementary Fig. 1e and Methods). We concluded that the LiP-based approach can be applied to study osmolyte effects on thermal stability.

We applied our optimized approach to an *E. coli* lysate, clustered the resulting peptide thermal profiles and observed that the resulting 20 clusters could be visually grouped into peptides with increasing intensity (purple; 40% of peptides), decreasing intensity (green; 42% of peptides) and nonmonotonic behavior (orange; 18% of peptides; Fig. 1c). Half-tryptic and fully tryptic peptides showed a similar profile distribution (Fig. 1d).

Based on known PK activity and cleavage preferences^43,46^, we interpret the observed fully tryptic profiles as follows (Fig. 1e). In the folded state, peptide bonds within tightly folded regions are typically poorly accessible to PK, but flexibility and accessibility increase after unfolding. Thermal profiles with decreasing fully tryptic peptide intensity (green) indicate increasing proteolytic accessibility and thus might be explained by protein unfolding. Profiles with increasing fully tryptic peptide intensity (purple), by contrast, indicate decreasing proteolytic accessibility and may therefore represent temperature-induced protein aggregation. Nonmonotonous profiles (orange) would represent a combination that is initial unfolding followed by aggregation. The exact opposite profiles are expected for half-tryptic peptides (Supplementary Fig. 1f). Our interpretation is consistent with the fact that *E. coli* proteins previously reported not to undergo temperature-induced precipitation^47^, defined here as nonprecipitating proteins, were enriched (*P* = 8.9 × 10^−28^, Fisher’s exact test) in peptides with a pure unfolding (green) profile (Fig. 1f and Supplementary Fig. 1g for half-tryptic peptides) relative to precipitating proteins. Overall, our data indicate that the proteome response to thermal denaturation is complex and that thermal profiling by LiP–MS could monitor events beyond protein unfolding alone.

### Osmolytes have a global effect on protein thermal stability

We applied the improved LiP–MS pipeline to monitor the effects of a panel of osmolytes on protein thermal stability across the proteome. We chose osmolytes from four chemical groups (sugars (glucose and trehalose), methylamines (TMAO and betaine), polyols (glycerol) and amino acids (proline)) with the goal of elucidating both general and osmolyte-specific effects. We used concentrations of osmolytes (1 M except 0.5 M for trehalose) consistent with those reported under stress conditions in cells^48,49^ and previously used in vitro^6–11^. The addition of osmolytes induced shifts in the profiles but mostly did not alter their shape (Supplementary Fig. 2a).

**Fig. 2:**
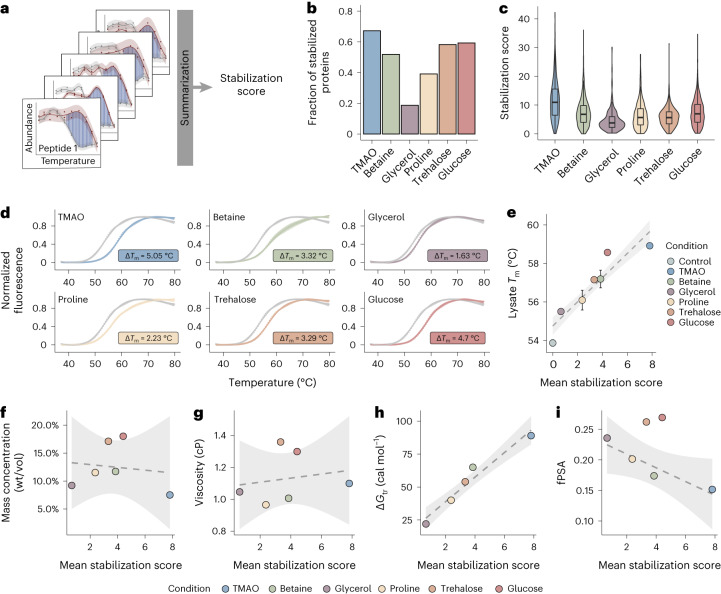
Osmolytes have a global effect on protein stability. **a**, Overview of the analytical procedure. A peptide-level score was calculated by summing the distances (blue) between thermal profiles in the absence (gray) and presence (red) of osmolyte at temperature values where confidence intervals do not overlap. Peptide-level scores were then combined after correcting for peptide length and overlap into a stabilization score for each protein (Supplementary Fig. 2c and Methods). **b**, Fraction of stabilized proteins out of all detected proteins for each osmolyte. **c**, Distribution of stabilization scores for proteins significantly stabilized by each osmolyte. Horizontal lines define the median, and boxes define the 25th and 75th percentiles; whiskers represent the maximum and minimum values. Each box plot represents the stabilization scores of stabilized proteins (205 proteins for glycerol, 791 for glucose, 885 for trehalose, 702 for betaine, 423 for proline and 733 for TMAO) calculated based on two LiP–MS replicates per temperature. **d**, Melting curves for *E. coli* cell lysate measured by DSF under control conditions (gray) and after the addition of osmolytes. Error bars show mean ± s.d. (*n* = 5 replicates per condition). **e**, Linear regression between DSF-measured lysate melting temperatures and mean stabilization scores for all detected proteins. Error bars show mean ± s.d. (*n* = 5 replicates). The shaded area represents the confidence interval of the linear fit (dashed line). **f**–**i**, Linear regression between mean stabilization score and osmolyte mass concentration (wt/vol; **f**), osmolyte viscosity at 25 °C (**g**), transfer free energy (*G*_tr_) for backbone model substrate transferred from water to 1 M osmolyte^8^ (**h**) and fraction polar surface area^8^ (fPSA; **i**). Transfer free energy for glucose was not available in the literature. In all cases, the shaded area represents the confidence interval of the linear fit (dashed line); cP, centipoise.

We then assessed thermal stabilization by quantifying any shift in the peptide profiles after the addition of an osmolyte and summarizing peptide-level information into a protein-level stabilization score (Methods and Fig. 2a). At a peptide- and protein-level false discovery rate (FDR) of <0.05 (Supplementary Fig. 2b,c and Methods), all osmolytes stabilized a large fraction of detected proteome based on scores obtained for >1,000 proteins under each condition (Fig. 2b and Supplementary Data 1). TMAO stabilized the largest number of proteins (67.2% of detected proteins) and showed the strongest stabilization, whereas glycerol at the same concentration stabilized the fewest proteins (18.7%) and showed the weakest stabilization (Fig. 2c).

To validate these results, we quantified the effect of osmolytes on the melting temperature of a lysate by differential scanning fluorimetry (DSF). The DSF-measured melting temperature of an untreated *E. coli* lysate (54.07 ± 0.14 °C; Supplementary Fig. 2d) was close to that (54.92 ± 0.06 °C) reported for *E. coli* cytosolic proteins by thermal protein profiling (TPP)^47^, indicating that the approach is applicable to complex biological extracts. At the same concentrations used in our LiP–MS experiment, all osmolytes significantly increased the lysate melting temperature, with TMAO showing the strongest stabilization and glycerol the weakest (Fig. 2d), matching the LiP results. We observed a good correlation between DSF-determined melting temperatures of osmolyte-treated lysates and the mean LiP–MS stabilization score (Fig. 2e), thus validating our stabilization score. Lysate concentration/dilution had minimal effects on the results (Supplementary Fig. 2e). Together, our data indicate that several classes of osmolytes have a global stabilizing effect on the proteome.

### Mechanism of global protein stabilization by osmolytes

We next investigated the different existing hypotheses on how osmolytes stabilize proteins, including stabilization effects driven by crowding, viscosity, preferential exclusion and direct binding. We observed no correlation between stabilization score and either osmolyte mass concentration (Fig. 2f) or viscosity (Fig. 2g). Further, equalizing the viscosities of added TMAO, betaine, proline and glycerol to that of 0.5 M trehalose or 1 M glucose did not equalize the stabilization efficiency (Supplementary Fig. 2f,g). This suggests that osmolyte-dependent thermal stabilization in cell lysates is not primarily due to effects on crowding and viscosity.

The preferential exclusion theory postulates that osmolytes form unfavorable interactions with the protein backbone such that folded protein states are favored in the presence of osmolytes^8,15,39^. The extent of protein stabilization by preferential exclusion can be approximated by the free energy change after transfer of a model protein backbone from water to 1 M osmolyte^8^. We observed a good correlation (*R*^2^ = 0.93) between our mean stabilization score and previously calculated transfer free energies for each osmolyte (Fig. 2h)^8^, demonstrating that preferential exclusion can explain the osmolyte stabilization effect in cell lysates^8,15,39^. In addition, according to this theory, differential osmolyte stabilization effects are explained by differences in the fractional polar surface areas of osmolytes. Indeed, for nonsugar osmolytes, we observed a high negative correlation between fractional polar surface areas and the mean stabilization score of lysates (Fig. 2i; *R*^2^ = 0.85), consistent with previous data for a single purified protein^8^ and further supporting the model of osmolyte-dependent protein stabilization by preferential exclusion.

### Biophysical features of stabilized proteins

**Fig. 3:**
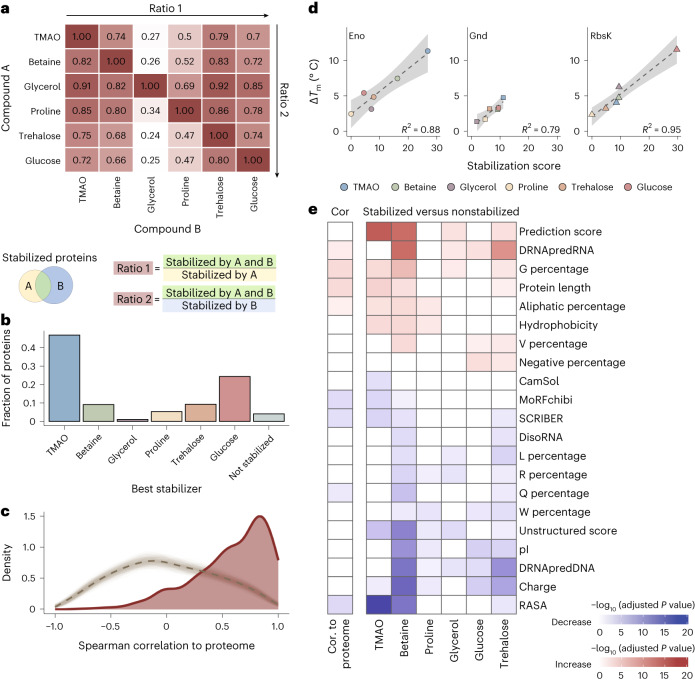
Biophysical features of osmolyte-stabilized proteins. **a**, The heat map shows pairwise overlap of osmolyte-stabilized proteins. Ratio 1 and ratio 2 are calculated as indicated. **b**, Fraction of proteins stabilized most strongly by each osmolyte out of all proteins detected in all six datasets. **c**, Density estimate distributions of the Spearman correlation coefficient between the stability score per protein and the mean stability score across the lysate (red line) or a randomized mean stability (dashed gray line) obtained from 1,000 repeated calculations (light gray lines; appears as a gray band). **d**, Linear regression between DSF-calculated ∆*T*_m_ and protein stabilization score for the indicated purified proteins. The shaded area represents the confidence interval of the linear fit (dashed line). Error bars (smaller than point size) show the standard deviation (*n* = 4 replicates). **e**, Heat map showing the most significantly different features (adjusted *P* value of <0.0001 in at least one comparison) between proteins with high correlation and low correlation of the stabilization score with that of the global proteome (first column, Cor) or between proteins significantly stabilized and those not stabilized by the indicated individual osmolytes. The color indicates whether the feature is higher (red) or lower (blue) in proteins with high correlation (first column) and in stabilized proteins (other columns) than in the rest. Feature significance was determined by two-sided *t*-test followed by correction by multiple hypothesis testing (Benjamini–Hochberg). Other tested features are shown in Supplementary Fig. 3. The term ‘X percentage’ refers to the fraction of the indicated amino acid. See Methods for the calculation or prediction of features.

There were large overlaps in the set of stabilized proteins between most osmolyte pairs (Fig. 3a). For instance, TMAO, glucose and trehalose all stabilized >70% of the proteins that each other osmolyte did, although this fraction was much smaller for glycerol (<30%). Further, of the 860 proteins that we reproducibly measured under all conditions, about half were stabilized by four or more osmolytes, and 11.5% of proteins were stabilized by all osmolytes (Supplementary Fig. 3a); note that because these analyses do not take false-negative rates into account (that is, sensitivity), these numbers are likely underestimates. Only 36 proteins were not stabilized by any osmolytes (Fig. 3b), and, interestingly, this set was enriched in proteins involved in stress responses and clearance of reactive oxidative species (*P* < 0.001, Fisher’s exact test). The relative strength of stabilization (Fig. 3b) indicates that TMAO is the best stabilizer for about half of the proteins and glucose for around 25% of the proteins. These results suggest that the tested osmolytes stabilize similar sets of proteins, but stabilization strength is osmolyte dependent.

Many, but not all, individual proteins showed a high correlation of stabilization score with the mean proteome score (Fig. 3c and Supplementary Data 1). DSF-derived melting temperatures for three purified proteins (6-phosphogluconate dehydrogenase (Gnd), enolase (Eno) and ribokinase (RbsK)) showed a good correlation (*R*^2^ > 0.79) with the LiP–MS-derived score in lysate (Fig. 3d and Supplementary Fig. 3b), showing that osmolyte stabilization is protein specific and also validating the stabilization score at the individual protein level. Although preferential exclusion is a good predictor of bulk proteome stabilization by osmolytes, we asked whether specific biophysical protein features are enriched in specific osmolyte-stabilized proteins. We compared 54 physicochemical and biochemical characteristics based on both protein sequence and structure (see Methods and Supplementary Fig. 3 for a full list) between the stabilized and nonstabilized set of proteins for each osmolyte as well as between those proteins that were well (Spearman correlation of >0.5) and poorly (Spearman correlation of <0.5) correlated in their stabilization score with the global proteome (Fig. 3e). Although the detailed patterns for different osmolytes varied, the data showed some general trends (Fig. 3e and Supplementary Fig. 3d). Many osmolytes preferentially stabilized proteins with lower charge and lower pI. Glucose and trehalose preferentially stabilized proteins with a higher percentage of negatively charged amino acids. TMAO, betaine and trehalose preferentially stabilized larger proteins with low relative accessible surface areas (RASAs) and low numbers of unstructured regions, indicating stabilization of globular compact proteins and again supporting a preferential exclusion mechanism for these osmolytes. Likewise, proteins that were stabilized in the same way as the global proteome (Spearman correlation of >0.5) were large, compact globular proteins with low RASAs and unstructured scores. Overall, this analysis confirms that a large fraction of proteins, in particular those with a compact globular structure, was stabilized through preferential exclusion, although additional factors may explain the stabilization of other proteins.

### Stabilization of multidomain proteins

**Fig. 4:**
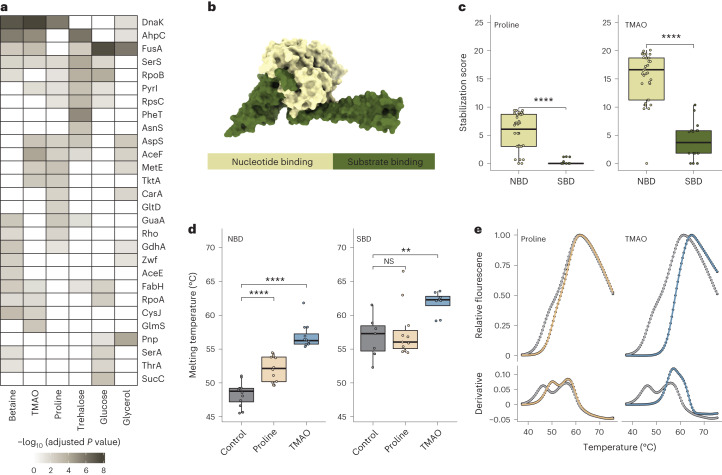
Stabilization of multidomain proteins. **a**, Heat map showing multidomain proteins with domains differentially stabilized by the indicated osmolytes (adjusted *P* value of <0.05; as determined by two-sided *t*-test and Benjamini–Hochberg correction). **b**, Structure of the chaperone DnaK (PDB ID: 4JNE) with the nucleotide binding domain (light green) and substrate binding domain (dark green). **c**, Distribution of the indicated osmolyte stabilization scores for amino acids mapping to the nucleotide-binding domain (NBD) and substrate-binding domain (SBD) of DnaK. Horizontal lines define the median, and boxes define the 25th and 75th percentiles; whiskers represent the maximum and minimum values. Significance was determined using two-sided Wilcoxon tests; ****P < 0.0001. Each dot represents an amino acid level stabilization score calculated based on two LiP–MS replicates per temperature. **d**, Distribution of melting temperatures derived from peptides mapping to the indicated domains of DnaK in the presence and absence of the indicated osmolytes. Each point represents one peptide curve measured in duplicate (*n* ≥ 7 peptides per condition). Box plots are as in **c**; **P < 0.01; ****P < 0.0001; NS, not significant. **e**, Protein melting curves measured with DSF and the first derivative of the melting curve for purified DnaK are plotted for control (gray), proline (yellow) and TMAO (blue).

To further elucidate osmolyte mechanisms of action, we took advantage of the peptide-level resolution of LiP–MS to ask whether osmolytes can stabilize single domains of multidomain proteins. For proteins with greater than one annotated domain and greater than or equal to three detected peptides per domain (*n* = 157 proteins), 28 proteins showed differential stabilization of domains (Fig. 4a). Most of these proteins showed domain-specific stabilization for more than one osmolyte, suggesting that this property was protein dependent and not osmolyte dependent. The strongest domain-specific effects were in the chaperone DnaK, and we explored this case further.

The nucleotide-binding domain and substrate-binding domain of DnaK (Fig. 4b) were differentially stabilized by TMAO, betaine, proline and glycerol, with better stabilization of the nucleotide binding domain and the biggest difference for proline and TMAO (Fig. 4c). Notably, the nucleotide binding domain showed a lower thermal stability than the substrate binding domain in the absence of added osmolytes (Fig. 4d), with the difference reduced after the addition of TMAO or proline. We validated these findings using DSF on purified DnaK (Fig. 4e). As in LiP–MS, DSF showed a melting behavior with two transitions in the absence of added osmolytes, and this behavior was lost after the addition of TMAO or proline. Our data show that osmolytes can preferentially stabilize individual domains of multidomain proteins and that this can reduce stability differences between domains.

### Direct binding to proteins causes strong stabilization

Protein stabilization could also be affected by direct protein–osmolyte binding, and we observed this effect for known binding partners of betaine, proline and glucose (Supplementary Fig. 4a). The effect was osmolyte specific (Supplementary Fig. 4b), indicating that this set of proteins is not generally better stabilized. For glucose, known monosaccharide-binding proteins were also stabilized (Supplementary Fig. 4c), in line with the monosaccharide binding promiscuity reported for some sugar kinases, for example, fructokinase^50^. Both glucokinase, which phosphorylates glucose, and fructokinase were more strongly stabilized by glucose than by other osmolytes (Supplementary Fig. 4d).

Interestingly, we also observed strong stabilization of RbsK by glucose, which is not known to bind this osmolyte, and to a lesser extent by glycerol (Supplementary Fig. 4d). We therefore asked whether the stabilization of RbsK was due to currently unknown binding of glucose to the ribose-binding site. Melting temperatures of purified RbsK in competition experiments between increasing ribose concentrations and added osmolyte suggest that glucose and glycerol stabilized RbsK through binding to the ribose-binding site because high ribose can compete away their stabilizing effect (Supplementary Fig. 4e). By contrast, stabilization by TMAO rose sharply with increasing ribose, which is known to cause compaction of RbsK^51^, and therefore suggests that TMAO acts via a preferential exclusion effect.

We next asked whether osmolytes affected protein thermal stability by altering protein structure more generally, either via currently unknown binding events or other indirect effects such as structure compaction. We focused on the first two temperatures (37 °C and 40.5 °C) in the thermal gradient, at which most proteins are not expected to unfold, and identified proteins with altered proteolytic susceptibility after osmolyte addition. We previously showed that this approach enables the global detection of metabolite–protein binding events (LiP-Smap)^41^. More than 50% of stabilized proteins under all conditions (and >90% for trehalose and glycerol) did not show any structural alteration after addition of the osmolyte to the lysate (Supplementary Fig. 4f and Supplementary Data 2). Thus, osmolytes induce structural changes in some proteins, but such changes, including direct osmolyte binding, are not required for stabilization across the proteome.

### Role of osmolytes in aggregation

Osmolytes were previously shown to affect aggregation of specific proteins^52,53^, but their effects across the proteome are unclear. We therefore investigated the effects of osmolytes on protein aggregation across the proteome. As discussed earlier (Fig. 1d), decreasing protease accessibility in an LiP–MS thermal profiling experiment is likely to indicate protein aggregation. However, LiP data alone cannot distinguish between aggregation and other structural rearrangements that decrease susceptibility to proteolysis. We thus made use of TPP, which measures thermal stability by monitoring protein aggregation.

**Fig. 5:**
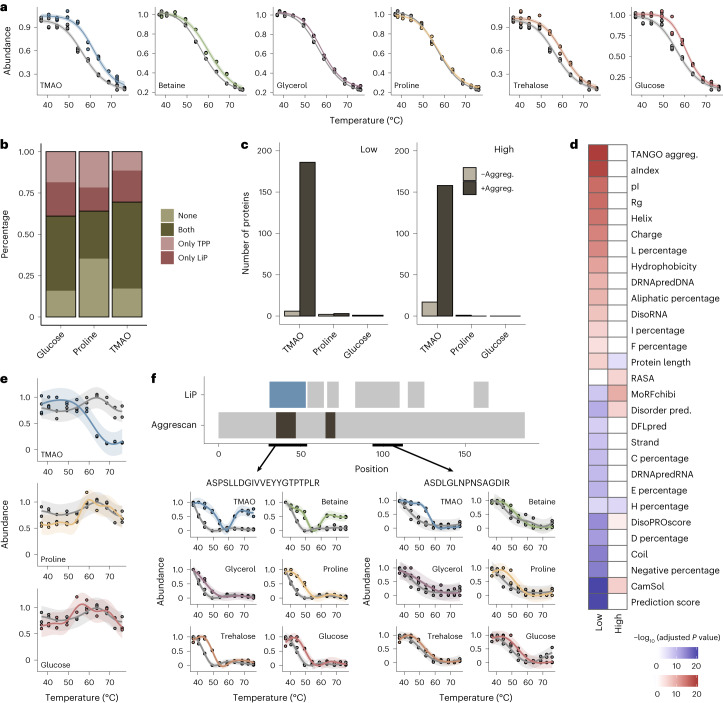
Osmolyte effects on protein aggregation. **a**, TPP-determined insolubility profile of *E. coli* lysate in the absence (gray) and presence (colors) of osmolytes. Plots show protein concentration in the soluble fraction scaled to the value at 37 °C. Shaded areas indicate the confidence interval of the fit. **b**, Comparison of protein stabilization analysis by LiP–MS and TPP. The plot shows the fraction of proteins reported stabilized by both methods (both), neither method (none) and one method (only LiP and only TPP). Only proteins defined as precipitators in TPP and detected in both datasets are included. **c**, Number of proteins significantly changed in abundance in the soluble fraction at low (37 °C and 42 °C) and high (68.2 °C, 72.5 °C and 76 °C) temperatures in TPP in the presence and absence of the indicated osmolytes. Significance was calculated separately for proteins with increased aggregation (+Aggreg.; fold change of <−1; dark gray) and decreased aggregation (–Aggreg.; fold change of >1; light gray) in the presence of osmolyte; adjusted *P* value of <0.05 (data were analyzed by two-sided *t*-test with a Benjamini–Hochberg correction). **d**, Heat map showing the most significantly different features (adjusted *P* value of <0.01; data were analyzed by *t*-test with a Benjamini–Hochberg correction) between proteins with increased aggregation in TMAO at high or low temperature (as in **c**) and all other detected proteins. Color indicates higher (red) or lower (blue) features in the increased aggregation group. Rg, radius of gyration; aIndex, aliphatic index. **e**, TPP profile for Frr in the absence (gray) and presence of the indicated osmolytes. The shaded area indicates the confidence interval of the fit. **f**, LiP–MS thermal profiles for two representative peptides from Frr for the indicated osmolytes. The top plot shows peptide positions along the Frr protein sequence. Top track, all detected peptides; blue, peptides with an increased aggregation profile under at least one condition; gray, peptides with no aggregation profile under any condition. Bottom track, predicted aggregation-prone regions (dark gray). The shaded area indicates the confidence interval of the fit.

We performed TPP under the same conditions as LiP–MS and probed the effect of osmolytes on thermal profiles. Insoluble aggregates typically appeared at higher temperatures in the presence of osmolytes than in the absence of osmolytes except for glycerol and proline, which had no effect, but none of the osmolytes prevented aggregation at the highest temperatures (Fig. 5a), when proteins are known to be unfolded^42,47^. More than 60% of detected proteins showed agreement between the LiP and TPP experiments for the stabilization effects of all three osmolytes (Fig. 5b, Supplementary Data 3 and Methods). In addition, both TPP and LiP identified TMAO as globally the best stabilizer, followed by glucose (Supplementary Fig. 5a). The good agreement between these two methods indicates that the upward shift in the temperature of aggregation in the presence of osmolytes occurs via stabilization of protein structure.

To monitor the effects on aggregation at both near-physiological and high temperatures, we focused on the lowest temperatures (37 °C and 42 °C) and the highest temperatures (69 °C, 72 °C and 76 °C) of the TPP data; for the latter set, most proteins will have lost their native structure. Proline and glucose did not affect protein aggregation. TMAO treatment yielded less aggregation of a few proteins (6/18 proteins at low/high temperatures; Supplementary Fig. 5b), which we confirmed using LiP–MS was via strong stabilization of protein structure (Supplementary Fig. 5c). Surprisingly, TMAO caused more aggregation of 186 and 158 proteins at the tested temperatures (Fig. 5c and Supplementary Data 3). At the lower temperatures, this set was predicted to have low solubility and be aggregation prone (TANGO score; Methods), suggesting that these were proteins on the edge of solubility. By contrast, TMAO promoted the high-temperature aggregation of proteins that were generally more soluble than the proteome (Supplementary Fig. 5d). In addition, these proteins had more predicted short disordered protein-binding regions^54^, higher RASAs and predicted disorder and were shorter than the rest of the measured proteome (Fig. 5d). They also had a lower percentage of histidines (Fig. 5d) and were enriched for periplasmic proteins (Supplementary Fig. 5e), which tend to unfold but not aggregate^55^. Our data thus indicate that TMAO promotes the aggregation of small, partially unfolded proteins that otherwise tend to remain soluble after unfolding.

To understand the effect of TMAO better, we focused on the ribosome recycling factor (Frr), which remained soluble across the entire temperature gradient but precipitated at higher temperatures in the presence of TMAO (Fig. 5e). This effect was also observed by LiP–MS, which further provided peptide-level information on Frr structural changes (Fig. 5f). The LiP–MS unfolding profiles reported that several osmolytes stabilized Frr. For the peptide ASPSLLDGIVVEYYGTPTPLR (amino acids 32–52 (blue region), predicted to be aggregation prone; see Methods), the profile changed from unfolding to nonmonotonous after the addition of TMAO or betaine, indicating that the protein precipitated at high temperatures in the presence of these osmolytes. Purified Frr behaved similarly, suggesting that the effects of TMAO and betaine are direct rather than via chaperones or binding partners (Supplementary Fig. 5f). Further, because TMAO stabilized the Frr structure as shown with both LiP–MS (Fig. 5f) and circular dichroism (Supplementary Fig. 5g), we concluded that TMAO promotes aggregation only after the protein has unfolded.

In conclusion, the addition of osmolytes caused an upward shift in the aggregation temperature of most proteins due to osmolyte stabilization of protein structure. However, TMAO in particular promoted the aggregation of subsets of proteins.

### Osmolyte effects on the human proteome

To assess whether our observations are generalizable and to investigate the thermal behavior of intrinsically disordered proteins (IDPs), we analyzed in situ osmolyte effects on the human proteome. IDPs are estimated to comprise 33% of the human proteome versus 4.2% in *E. coli*^56^ and are in several cases associated with human disease^57^. We thus studied both the in situ thermal unfolding behavior and osmolyte effects of this interesting set of proteins. We focused our analysis on TMAO because it promoted aggregation of proteins with higher predicted disorder in *E. coli* while strongly stabilizing globular proteins; trehalose was also included for comparison.

**Fig. 6:**
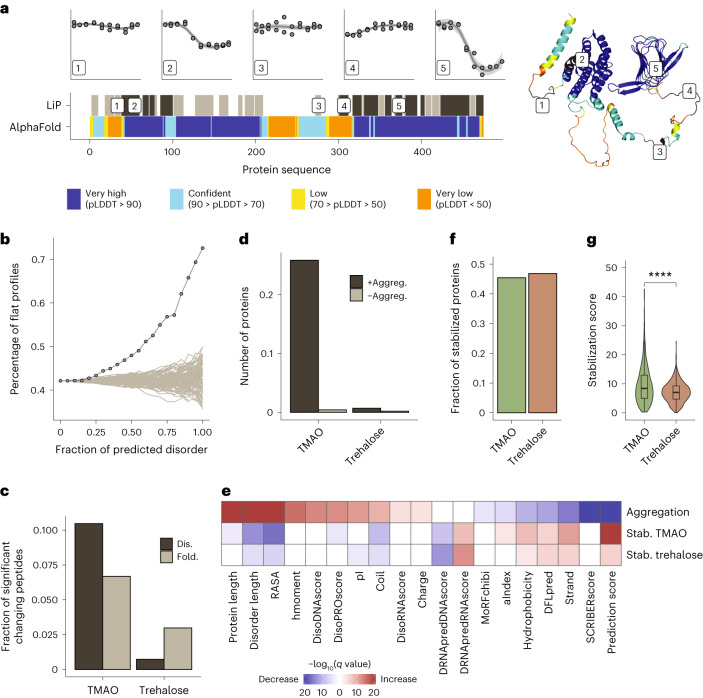
Osmolyte effects on the human proteome. **a**, Analysis of predicted disorder in regions of CAP-1 with different thermal melting behavior. Plots (top) show thermal profiles of five example peptides mapping to indicated regions along the protein sequence. The top barcode (LiP) shows all peptides (dark gray) with a measurable thermal melting profile out of all detected peptides (light gray) along the protein sequence. The lower barcode shows the AlphaFold pLDDT prediction score^71^; very low scores typically correspond to disordered protein regions. The AlphaFold-predicted structure is shown (right), with peptides annotated. **b**, Percentage of peptides with flat profiles (lines with dots) for proteins with increasing fractions of predicted disorder. All detected peptides/proteins are plotted. Gray lines show tests in which protein disorder was randomized. **c**, Fraction of peptides that show a structural change following osmolyte treatment relative to control out of all detected peptides calculated separately for peptides predicted to be folded (Fold) or disordered (Dis). **d**, Number of proteins with increased (+Aggreg.) or decreased (–Aggreg.) aggregation after the addition of osmolytes to HEK293T cell lysates at 37 °C. **e**, Biophysical features of human proteins affected by osmolytes. The heat map shows significantly enriched or depleted features for proteins that precipitate in the presence of TMAO versus those that do not precipitate (row 1, aggregation) or that are stabilized (Stab.) in the presence of the indicated osmolytes versus nonstabilized proteins (rows 2 and 3). *P* values were determined by two-sided *t*-tests followed by Benjamini–Hochberg multiple testing correction. hmoment, hydrophobic moment. **f**, Fraction of proteins stabilized in HEK293T cell lysates in the presence of TMAO and trehalose. **g**, Distribution of stabilization scores for proteins significantly stabilized by TMAO (899 proteins) and trehalose (948 proteins) based on two LiP–MS replicates per temperature. Horizontal lines indicate the median, boxes indicate the 25th and 75th percentiles, and whiskers indicate the maximum and minimum values. Significance was determined by two-sided Wilcoxon tests; ****P < 0.0001.

We first examined the thermal unfolding behavior of the human proteome in the absence of added osmolytes. Consistent with our previous observations on α-synuclein^42^, protein regions with high predicted disorder tended to show flat rather than thermal unfolding (that is, sigmoidal) profiles. For instance, the CAP-1 protein showed clear thermal unfolding profiles for regions predicted to be folded (peptides 2 and 5), whereas peptides mapping to predicted disordered regions (peptides 1, 3 and 4) showed a flat profile not affected by temperature (Fig. 6a). Across the proteome, proteins with a high fraction of predicted disorder had significantly more peptides with a flat profile than folded proteins (Fig. 6b; flat profiles were defined as those with absolute log_2_ (fold change) values of <0.5 between the minimum and maximum peptide intensity values). Similarly, peptides originating from regions with high predicted disorder had a significantly flatter thermal profile than peptides from regions with low predicted disorder, which are more likely to be folded (Supplementary Fig. 6a). Overall, 62.6% of peptides predicted to be disordered (pLDDT score of <50) had a flat thermal profile (Supplementary Data 4). Thus, our data enable the definition of regions of the human proteome (*n* = 1,864 peptides, corresponding to 727 proteins) that have flat thermal unfolding profiles and are predicted to be intrinsically disordered, providing evidence for intrinsic disorder in situ. Interestingly, the remaining 37.4% of peptides predicted to be disordered showed evidence of thermal unfolding in cell lysates (Supplementary Fig. 6b); an example is peptides from the protein PCBP2 (Supplementary Fig. 6c). These data suggest that a substantial fraction of predicted disordered regions may fold, bind other molecules or both in cell lysates (see Discussion).

We then examined the effects of TMAO and trehalose on human proteins. We first asked how osmolytes affect the structure of folded and disordered regions of human proteins by analyzing LiP–MS data from the first two temperatures (37 °C and 40.5 °C) of our thermal profiling gradient. As in *E. coli*, only a very small fraction of proteins showed structural effects of TMAO or trehalose, but, interestingly, we observed different effects on folded and disordered regions. TMAO affected around 10% of disordered regions, whereas folded regions were less affected. By contrast, trehalose showed almost no structural change in disordered regions, and its overall effect was smaller (Fig. 6c). Neither TMAO nor trehalose changed the fraction of proteins with flat thermal profiles in a human cell lysate, consistent with these osmolytes having no strong global structural effect (Supplementary Fig. 6d). These patterns could also be exemplarily seen for the oncogenic protein EWS, an IDP associated with Ewing sarcoma and other cancers^58^ (Supplementary Fig. 6c).

We further analyzed how TMAO and trehalose affect aggregation of human proteins. At physiological temperature (37 °C), TMAO, but not trehalose, promoted aggregation of 25% of human proteins (Fig. 6d and Supplementary Data 4). This set was enriched in large disordered proteins with low β-sheet content, low propensity to bind proteins (SCRIBER score) and low AlphaFold prediction score (Fig. 6e), indicating that TMAO promotes the aggregation of large disordered human proteins. This set included the human disease proteins p53, huntingtin and EWS, all of which precipitated in the presence of TMAO but not trehalose (Supplementary Fig. 6e).

Finally, we focused on the subset of around 2,000 human proteins for which we could derive thermal unfolding profiles, which was enriched in globular proteins as expected (Supplementary Fig. 6f). Both TMAO and trehalose stabilized more than 40% of the analyzed proteins (Fig. 6f and Supplementary Data 4), and, as in *E. coli*, the effect of TMAO was stronger (Fig. 6g). The stabilized proteins were enriched in globular proteins with high AlphaFold prediction score, high β-sheet content, low RASA and low predicted disorder (Fig. 6e), in line with the set of proteins preferentially stabilized by TMAO in *E. coli* and supporting that human proteins are also stabilized through preferential exclusion. This analysis also highlights the dual effect of TMAO to strongly stabilize globular proteins and promote aggregation of large disordered proteins.

Overall, our analysis of the human proteome shows that the stabilizing effects of osmolytes on proteins are general, supports the existence of IDP regions in situ, highlights interesting instances of potential in situ structure in disordered regions and demonstrates differential effects of TMAO on disordered and globular proteins.

## Discussion

We have extended LiP–MS-based thermal profiling into a robust method to study proteome thermal stabilization in situ. This approach allowed us to separately analyze the effects of osmolytes on protein thermal unfolding, native structure and aggregation, thus going beyond the simple identification of osmolyte-stabilized proteins and shedding light on mechanisms of osmolyte action. Our approach further allowed for the assessment of whether direct binding is required for protein stabilization. We report a global analysis of osmolyte effects in a cell-like environment, revealing that osmolyte-dependent protein thermal stabilization is a widespread phenomenon in situ. Our approach can be applied to any small molecule with proposed stabilizing effects. Analysis of the human proteome shows that LiP–MS can be used to study the behavior of intrinsically disordered regions in situ.

We revealed biophysical characteristics of the stabilized proteome as well as mechanisms of osmolyte action. Our data are consistent with a preferential exclusion mechanism for most protein–osmolyte pairs^8^, although observations for several individual proteins did not match the predictions of this theory. This might be because the predictions are based on in vitro models and do not consider protein–protein and protein–metabolite interactions that can influence thermal stabilization^59^. Indeed, our data show an example of such a case, where ribose binding to the protein RbsK increased the stabilization efficiency of TMAO. We identified many other candidate osmolyte binding proteins, although the structure of most proteins did not change after the addition of osmolytes. Most osmolytes caused an upward shift in the temperatures at which proteins aggregate, most probably by stabilizing protein structure. However, the osmolyte TMAO was an exception; it both thermally stabilized many proteins with relatively strong effects and promoted aggregation in both *E. coli* and human proteomes. Intriguingly, our data suggest that TMAO stabilizes globular proteins while promoting the aggregation of disordered proteins, potentially helping to explain opposing reports in the literature^32,52,60^.

Intriguingly, in nature, TMAO is typically present in contexts in which there are also high levels of urea, a known protein denaturant^61,62^. For instance, chondrichthyans (sharks, rays and skates) accumulate high concentrations of urea but also TMAO in a ratio of 2:1 (urea:TMAO)^63^. It is plausible that TMAO stabilization of most proteins is adaptive in such a context and also that urea prevents the potentially detrimental TMAO-dependent aggregation in some proteins. Such effects have been observed for the IDP α-casein, for which urea prevents TMAO-induced protein aggregation^64^.

Our observation of good overlap between proteins stabilized by different osmolytes helps explain their interchangeability in protecting microorganisms following heat stress. However, use of osmolytes is not uniform; for instance, TMAO is mostly found in marine organisms^65,66^, whereas plants largely use proline and betaine^67^. Selection of specific osmolytes by evolution may also have been driven by other factors, for instance, compatibility with enzyme function, availability, cost of uptake or production or other osmolyte functions.

Our analysis of the human proteome provides global in situ evidence for protein intrinsic disorder but also evidence for folding in protein regions corresponding to more than one-third of peptides predicted to be disordered. We note that it is challenging, however, to define appropriate thresholds for foldedness (of a protein region) and for flatness (of a peptide profile), both of which are needed for such analyses. In addition, disorder prediction algorithms are unlikely to be perfect. Nevertheless, our data suggest that a fraction of proteins predicted to be intrinsically disordered may take on unexpected structure in the cellular context, which may be due to protein–protein interactions, post-translational modifications or other consequences of the cellular milieu. LiP–MS thermal profiling could be an exploratory tool both to study such effects and to identify proteins or protein regions that are disordered within cell lysates. Our analysis, with peptide-level resolution, enabled us to probe the behavior of IDRs within a protein sequence and expands our previous protein-level analysis of IDPs^42^.

Finally, our study could help identify new stabilizers. We showed that DSF on cell lysates can accurately predict global stabilization effects of osmolytes, which could form the basis of a new stabilizer screening pipeline. DSF on a complex protein mixture could be used first to select promising molecules that show global effects, followed by LiP–MS on selected hits to profile their effects on specific proteins or protein groups and to study mechanisms. Interestingly, stabilization through direct binding tends to cause a larger shift at the same osmolyte concentrations than stabilization through preferential exclusion. This suggests that, purely for stabilization purposes, small molecules that directly bind to the protein should be favored, although a balance between stabilization and retention of protein activity must be found. Further, differential effects of an osmolyte on different proteins, as we have observed for TMAO, must be considered. Our dataset showed that, although osmolytes are broadly interchangeable, for most proteins, either TMAO or glucose was the best stabilizer; these data can be further used to specifically identify which stabilizer is best for which protein or to study combinatorial effects. Last, our data could drive machine learning approaches to predict protein stabilization by a given osmolyte^68^.

A few caveats must be considered. First, because the experiments were performed in cell lysates, the effects may not entirely reflect the situation within intact cells. Second, the concentrations of osmolytes that we used are close to cellular values^48,49^ but may be high for biotechnological applications. This could be circumvented by designing polymers with osmolyte active groups to reach a desired effect at lower concentrations^69^. Third, although LiP–MS provides information-rich datasets of structural changes, mechanistic interpretation of these changes can be challenging; as shown here, these can be tackled by combination with an orthogonal approach such as TPP^47^. Finally, our data do not report on important additional factors, such as osmolyte toxicity, which must be considered when selecting an optimal stabilizer. For example, increased TMAO concentrations have been associated with increased risk of cardiovascular disease^70^.

Increasing the thermal stability of enzymes and protein-based drugs is a major challenge in biotechnology. Our study shows the potential of small molecules as protein stabilizers because we found that specific osmolytes or combinations thereof can stabilize most proteins. Further, we showed that our thermal profiling approach can differentiate between osmolyte mechanisms of action and can help identify molecules that increase thermal stability or counteract irreversible aggregation. This global and in situ analysis of osmolyte action has shown which effects of osmolytes are protein specific and which are global and has allowed us to extrapolate observations for purified proteins into general theory. Our study advances the biophysical and biological understanding of osmolyte mechanism of action and provides practical insight into the development of better protein stabilizers.

## Methods

### *Escherichia coli*

*E. coli* strain BW25223 K12 (ref. ^72^) was grown in M9 minimal medium^73^ containing 2 g l^–1^ glucose at 37 °C and 800 rpm. A single colony was picked from a fresh plate to inoculate Luria–Bertani (LB) complex medium and incubated for 6 h at 37 °C with constant shaking. LB cultures were diluted 1:100 with M9 minimal medium and incubated overnight. Overnight cultures were diluted to an optical density at 600 nm (OD_600_) of 0.05 and collected when the OD_600_ reached 0.8.

### HEK293T cells

HEK293T cells were cultured in DMEM (Gibco, 41965039) supplemented with 10% fetal bovine serum and 1% penicillin/streptomycin and passaged before confluency by detachment with 0.25% trypsin, followed by washing in PBS (pH 7.4; Gibco, 10010015). To store the pellets, cells were centrifuged at 900*g* for 2 min. PBS was removed, and the pellets were stored at –20 °C until further use.

### Cell lysate preparation

#### *E. coli*

Frozen cells were thawed on ice and resuspended in lysis buffer (20 mM HEPES, 150 mM KCl and 10 mM MgCl_2_, pH 7.5). Acid-washed glass beads were added, and the cells were lysed with three rounds of vortexing for 30 s, followed by a 4-min incubation at 4 °C. The lysates were centrifuged (20,000*g*, 15 min, 4 °C) to remove cell debris. The supernatants were transferred to a fresh tube, and protein concentration in the lysates was determined by bicinchoninic acid assay (BCA Protein Assay Kit, Thermo Scientific).

#### HEK293T cells

Cell pellets were resuspended in 300 μl of LiP buffer (20 mM HEPES (pH 7.5), 150 mM KCl and 10 mM MgCl_2_) and lysed using a pellet pestle (Argos Technologies) in ten cycles of 10 s of homogenization and a 1-min pause on ice. The lysate was cleared by centrifugation at 15,000*g* at 4 °C for 10 min. The supernatants were transferred to a fresh tube, and protein concentration in the lysates was determined by bicinchoninic acid assay (BCA Protein Assay Kit, Thermo Scientific).

### Preparation of standard control sample of short peptides

*E. coli* cell lysates were mixed with urea (final concentration of 6 M) and reduced with 1,4-dithiothreitol (DTT; final concentration of 12 mM) for 30 min at 37 °C and subsequently alkylated with iodoacetamide (final concentration of 40 mM) for 45 min at room temperature. Samples were diluted with 0.1 M ammonium bicarbonate to a final urea concentration of 1 M and predigested with lysyl endopeptidase (Wako Chemicals) at an enzyme:substrate ratio of 1:100. After 4 h at 37 °C, sequencing-grade porcine trypsin (Promega) was added at a 1:100 ratio, and samples were incubated overnight at 37 °C and 800 rpm. The digestion was stopped by the addition of formic acid to a final pH of less than 3. The peptide mixtures were loaded onto Sep-Oak tC18 cartridges (Waters), desalted and eluted with 80% acetonitrile. All peptide samples were evaporated in a vacuum centrifuge to dryness and resolubilized with lysis buffer (20 mM HEPES, 150 mM KCl and 10 mM MgCl_2_, pH 7.5) to a peptide concentration of 1 mg ml^–1^.

### Preparation of osmolyte stock solutions

All osmolytes were prepared from ultrapure powders in lysis buffer (20 mM HEPES, 150 mM KCl and 10 mM MgCl_2_, pH 7.5) at 2× final concentration. If necessary, the pH was adjusted to 7.5 with 10 M NaOH solution. Stock solutions were frozen at −20 °C until use.

### Limited proteolysis

Cell lysates were mixed with osmolyte stock solutions to a final protein concentration of 2 mg ml^–1^ and an osmolyte concentration of 1 M (0.5 M for trehalose) or with buffer as a control. Lysates were aliquoted into ten wells (50-μl aliquots, two replicates for each condition) and incubated at a temperature gradient (37–76 °C; exact temperatures: 37, 40.5, 44.4, 49.3, 54.1, 58.9, 63.8, 68.6, 72.5 and 76 °C) for 5 min. Afterward, PK from *Tritirachium album* (Sigma) was added to the protein extract at an enzyme:substrate ratio of 1:50, mixed well by pipetting with a larger volume (30 μl) and incubated for 1 min at the assigned temperatures. The digestion was stopped by heating the sample for more than 4 min at 99 °C in a thermocycler and adding sodium deoxycholate (DOC) to a final concentration of 5%. The samples were then subjected to complete digestion under denaturing conditions as described below. For the temperature activity assay, the same protocol was used with predigested cell lysate (fully tryptic peptides at a concentration of 0.5 mg ml^–1^) instead of native cell lysate. To mimic different PK activities for analysis, the same protocol was used with three different enzyme:substrate ratios for PK (1:20, 1:50 and 1:100).

### Thermal proteome profiling

Cell lysates were mixed with osmolyte stock solutions to a final protein concentration of 2 mg ml^–1^ and an osmolyte concentration of 1 M (0.5 M for trehalose) or with buffer as a control. Lysates were aliquoted into ten wells (50-μl aliquots, two replicates for each condition) and incubated at a temperature gradient (37–76 °C) for 5 min. For the HEK293T cell precipitation assay, samples in triplicates, with and without osmolytes, were incubated at 37 °C for 5 min. After heating, samples were filtered by centrifugation at 800*g* for 5 min with a 0.2-μm PVDF membrane filter (Corning FiltrEX 96-well white filter plate). After centrifugation, 40 μl of flowthrough was transferred to a fresh plate and mixed with DOC to a final concentration of 5%. The samples were then subjected to complete digestion under denaturing conditions as described below.

### Tryptic digestion

Following LiP or filtration, protein fragments were reduced by incubation with DTT (final concentration of 12 mM) for 30 min at 37 °C and alkylated by incubation with iodoacetamide (final concentration of 40 mM) for 45 min at room temperature in the dark. Samples were diluted with 0.1 M ammonium bicarbonate to a final concentration of DOC of 1%. Proteins were digested overnight with lysyl endopeptidase (Wako Chemicals) and sequencing-grade porcine trypsin (Promega) at an enzyme:substrate ratio of 1:100 at 37 °C with constant shaking (800 rpm). The digestion was stopped by the addition of formic acid to a final concentration of 1% (pH < 3). Precipitated DOC was filtered by centrifugation at 800*g* for 5 min with a 0.2-μm PVDF membrane filter (Corning FiltrEX 96-well white filter plate). The peptide mixtures were loaded onto 96-well elution plates (Waters), desalted and eluted with 80% acetonitrile and 0.1% formic acid. After elution, peptides were dried in a vacuum centrifuge, resolubilized in 0.1% formic acid and analyzed by MS.

### Liquid chromatography–tandem mass spectrometry data acquisition

Peptides were separated on a Waters Acquity M-Class UPLC system on a 40 cm × 0.75 mm inner diameter column (New Objective, PF360-75-10-N-5) packed with 1.9-μm C18 beads (Dr. Maisch, Reprosil-Pur 120). Fractionation was achieved with a gradient of buffer A (0.1% formic acid, Carl Roth) and buffer B (99.9 % acetonitrile and 0.1% formic acid, Carl Roth): a linear gradient from 3% to 35% buffer B over 120 min, followed by 5 min with an isocratic constant concentration of 90% buffer B. The column was heated to 50 °C.

#### Data-dependent acquisition

Shotgun liquid chromatography–tandem MS data-dependent acquisition on the Orbitrap Fusion Lumos Tribrid mass spectrometer was performed solely for library generation. One replicate of each condition was selected randomly, and 2 μl from the sample was injected. MS^1^ spectra were acquired from 350 to 1,500 *m*/*z* at an orbitrap resolution of 120,000 with an automated gain control (AGC) target of 150% or 100-ms injection time. Precursors with an intensity exceeding 50,000 and a positively charged state between 2 and 5 were selected for data-dependent MS^2^ scans. A dynamic exclusion after a single occurrence for 30 s with a 10-ppm mass tolerance was applied. Selected precursors were isolated with a quadrupole and an isolation window of 1.2 *m*/*z*. Precursors were fragmented with high-energy collision-induced dissociation with a fixed collision energy of 27%. MS^2^ spectra were acquired at an orbitrap resolution of 30,000 and an automatically adapting scan range (minimum – precursor × charge + 10.0) and an AGC target of 200% or a dynamic injection time that automatically calculated the maximal time available.

#### Data-independent acquisition

For measuring peptide abundances, 2 μl of each sample was injected independently and was measured in data-independent acquisition (DIA) mode. The DIA MS method consisted of a survey MS^1^ scan from 350 to 2,000 *m*/*z* at a resolution of 120,000 with an AGC target of 50% or 100-ms injection time followed by the acquisition of DIA in 41 variable-width isolation windows^74^. Precursors were isolated by a quadrupole and activated high-energy collision-induced dissociation with a collision energy of 28%. DIA MS^2^ spectra were acquired with a scan range of 200 to 1,800 *m*/*z* at an orbitrap resolution of 30,000 with an AGC target of 200% or 54-ms injection time.

### Protein expression and purification

*E. coli* strains carrying plasmids encoding DnaK, Eno, Frr, RbsK and Gnd with an N-terminal His_6_ tag from *E. coli* were commercially obtained^75^. From the strain libraries, precultures were inoculated in LB broth with corresponding antibiotic. LB cultures (1.5 l) were inoculated with 15 ml of preculture and grown to logarithmic phase at 37 °C. Cultures were induced with 1 mM IPTG at an OD_600_ of 0.8 and were allowed to grow at 37 °C for 3 h with vigorous shaking.

Collected cells were suspended in 50 ml l^–1^ culture Buffer B (50 mM HEPES-KOH (pH 7.5), 150 mM NaCl and 10% glycerol) supplemented with 1 mM PMSF, 2 mM MgCl_2_, 50 U μl^–1^ DNase and 1× protease inhibitor cocktail (Sigma). Resuspended cultures were mechanically lysed by cell cracking with five passages at a chamber pressure of 12,000 psi. Cell debris was pelleted by centrifugation of lysed cells at 20,000 rpm from 30 min at 4 °C.

Soluble supernatant with 10 mM imidazole was applied to Ni-NTA agarose beads (Qiagen) with a column volume of 2.5 ml. Protein bound to the Ni-NTA agarose beads was washed with 10 column volumes of Buffer B supplemented with 20 mM imidazole. Proteins of interest were eluted from beads with 3 column volumes of Buffer B supplemented with 500 mM imidazole. An additional 1 mM EDTA was added to the eluted protein. Protein was further purified by gel filtration chromatography on a HiLoad 16/600 Superdex 75 pg column (Cytiva, formerly GE Healthcare) in Buffer S (50 mM HEPES-KOH (pH 7.5), 150 mM NaCl, 10% glycerol, 1 mM EDTA and 1 mM DTT). Purified DnaK, Eno, Frr, RbsK and Gnd were stored in Buffer S at −20 °C after snap freezing in liquid nitrogen. For various assays, the storage buffer was exchanged for assay buffer using PD MiniTrap G-25 columns (Cytiva, formerly GE Healthcare) by gravity flow.

### Differential scanning fluorimetry for whole-cell lysate

*E. coli* cell lysates were prepared as described above. Lysates were diluted to a final protein concentration of 1 mg ml^–1^ together with 10× Sypro Orange Gel stain (Thermo Fischer) and a buffer (control) or 1 M osmolytes (0.5 M for trehalose) in a total volume of 25 μl. Thermal scanning (25–95 °C, 0.5 °C min^–1^) was performed in quadruplicates using a CFX96 Touch Real-Time PCR Detection System (Bio-Rad) with the scan channel set to FRET. The data were fit using the thermal unfolding equation^76^, assuming a two-state unfolding model with the linear extension of before and after denaturation baseline. Because DSF is typically used with purified proteins, we first examined its performance on an untreated lysate using two different total protein concentrations and two different *E. coli* lysate preparations. To determine the reproducibility of the method, the experiment was performed on different days; different biological replicates were used and diluted to different protein concentrations (from 0.5 to 2 mg ml^–1^). Despite this, the lysate melting temperature was extracted reproducibly.

### Differential scanning fluorimetry for purified proteins

Proteins were purified as described above and diluted to a final protein concentration of 0.5 mg ml^–1^ together with 10× Sypro Orange Gel stain and a buffer (control) or 1 M osmolytes (0.5 M for trehalose) in a total volume of 25 μl. Thermal scanning (25–95 °C, 0.5 °C min^–1^) was performed in quadruplicates using a CFX96 Touch Real-Time PCR Detection System (Bio-Rad) with the scan channel set to FRET. For RbsK, the transitions were measured in the presence of increasing concentrations of ribose. The data were fit using the thermal unfolding equation^76^, assuming a two-state unfolding model with the linear extension of before and after denaturation baseline.

### Circular dichroism

Protein secondary structure and thermal transitions were measured using a Jasco J-715 spectropolarimeter (Brechbühler). Protein (300 μl) at a concentration of 0.2 mg ml^–1^ in buffer C (20 mM NaH_2_PO_4_.H_2_O/K_2_HPO_4_ (pH 7.5), 150 mM NaCl and 1 mM MgCl_2_) was measured in a high-precision cell quartz cuvette with a pathlength of 1 mm (Hellma Analytics). Secondary structure scans were continuously measured from 260 nm to 190 nm with a scanning speed of 20 nm min^–1^ and a total of five repetitions. The average ellipticity scans were buffer corrected. Ellipticity (*ϴ*) was then normalized to mean molar ellipticity in degree cm^–2^ dmol^–1^ (*MRW* = (*ϴ* × 100 × kDa)/(*c* × *d* × *N*)), where *N* is the number of amino acid residues, *d* is the cuvette pathlength in centimeters and *c* is the protein concentration in mg ml^–1^. Thermal unfolding of Frr was measured in the presence and absence of 1 M TMAO at 222 nm from 25 °C to 95 °C using a slope of 30 °C h^–1^. Changes in ellipticity as a function of temperature were fitted according to the single transition state model.

### Precipitation analysis for ribosome recycling factor

Frr was diluted to a final protein concentration of 0.3 mg ml^–1^ in lysis buffer or 1 M osmolytes (0.5 M for trehalose) in a total volume of 20 μl. Samples in triplicates were heated to 70 °C or 37 °C (as a control) in a thermoblock for 5 min and subsequently centrifuged at max speed using a table-top centrifuge. Protein concentration in the supernatant was determined in technical duplicates for each replicate using a Bradford assay (Thermo Fischer). Absorbance at 70 °C was divided by the average absorbance at 37 °C to calculate the percentage of remaining soluble protein.

### Thermal proteome profiling for whole-cell lysates

*E. coli* cell lysates were prepared as described above and diluted to a final protein concentration of 1 mg ml^–1^ together with lysis buffer or 1 M osmolyte (0.5 M for trehalose) in a total volume of 50 μl. The lysates were incubated at ten temperatures ranging from 37 °C to 76 °C for 5 min. After heating, samples were filtered by centrifugation at 800*g* for 5 min with a 0.2-μm PVDF membrane filter (Corning FiltrEX 96-well white filter plate). The flowthrough was collected in a 96-well plate, and protein concentration was determined using a Bradford assay (Thermo Fischer).

### Extended proteolysis assay

*E. coli* cell lysates were prepared as described above and diluted to a final protein concentration of 2 mg ml^–1^ with lysis buffer or 1 M osmolyte (final concentration) in a total volume of 50 μl. The lysates were incubated at 76 °C for 5 min. The sample was cooled to room temperature, and PK was added at an enzyme:substrate ratio of 1:50. After 5, 10 or 30 min, based on the assigned digestion time, samples were transferred to a cooled centrifuge and centrifuged for 15 min at maximum speed at 4 °C. Immediately after centrifugation, the supernatant was removed, and aggregates were resolubilized in 50 μl of 2% SDS by vortexing and sonication. Protein concentration of resolubilized proteins was determined by bicinchoninic acid assay (BCA Protein Assay Kit, Thermo Scientific). For time point 0 (no PK digestion), PK was not added to the sample.

### Detergent resolubilization assay

*E. coli* cell lysates were prepared as described above and diluted to a final protein concentration of 2 mg ml^–1^ with lysis buffer or 1 M osmolyte (final concentration) in a total volume of 50 μl. The lysates were incubated at 76 °C for 5 min, and the sample was cooled to room temperature and mixed (1:1) with increasing concentrations of sarcosyl in 1× PBS. After vortexing and sonication, samples were centrifuged (maximum speed, 15 min at 4 °C), and the concentration of protein in the soluble fraction was determined by bicinchoninic acid assay (BCA Protein Assay Kit, Thermo Scientific).

### Viscosity measurements

The viscosity of different 1 M osmolyte solutions was evaluated by measuring the apparent diffusion coefficient of standard 100-nm nanoparticles via dynamic light scattering, following a previously published protocol^77^. Briefly, the viscosity (*η*) of the solutions was calculated from measurements of the Stokes–Einstein equation,

$$\eta =\frac{{kT}}{6\pi {R}_{{\mathrm{h}}}D},$$

where *k* is the Boltzmann constant, *T* is the temperature, and *D* and *R*_h_ are the apparent diffusion coefficient and the hydrodynamic radius of the nanoparticles used as standard tracers, respectively. Dynamic light scattering measurements were performed on a Zetasizer Nano working in backscattering mode at 173°.

### Peptide- and protein-level quantification

The data were searched in Spectronaut version 14.5 (*E. coli*) and version 14.11 (human; Biognosys)^78^. Hybrid libraries for the tryptic control and LiP samples consisting of the corresponding data-dependent acquisition and DIA runs were created based on a Pulsar search using default settings except for the digest type, which was set to ‘semi-specific’, and the minimum peptide length, which was set to six. The data were searched against the UniProt fasta database (for *E. coli* strain K12, UP000000625, November 2020; for the human proteome, canonical fasta, downloaded March 2020). Targeted data extraction was performed in Spectronaut with default settings except for machine learning, which was set to ‘across experiment’, and data filtering, which was set to ‘Qvalue’. The FDR was set to 1% on the peptide and protein levels. From the LiP search, we exported peptide intensities from the tryptic control search (for TPP data) protein intensities.

### Fully tryptic and half tryptic peptides

PK cleavage within the sequence of an fully tryptic peptide often results in the detection of half-tryptic peptides that share either the N or the C terminus with the associated fully tryptic peptide. Our previous study only analyzed fully tryptic peptides that reported an altered accessibility to PK with temperature for the respective protein region^42^. In the current study, both fully tryptic and half-tryptic peptides were included in the analysis.

### Correlation analysis for fully tryptic and half tryptic pairs

Fully tryptic and half-tryptic peptides were matched by their assigned position in a protein. Half-tryptic peptides were matched with an fully tryptic peptide if the borders of the half-tryptic peptides were within the borders of the fully tryptic peptide. Pearson correlation was calculated between the peptide intensities of the matched pairs.

### Testing and correcting for condition effects on proteinase K activity

Left unadjusted, any effects of experimental conditions on PK activity could bias the analysis. We performed a protease activity assay using a mixture of more than 3,000 short (less than ten amino acids) peptides to evaluate PK activity independent of folding effects. We tested whether we observed significantly different digestion of these short peptides when LiP was performed at different temperatures or in the presence of osmolytes. To eliminate the peptides with potential secondary structure, we only focused on approximately 3,000 fully tryptic peptides that were at most ten amino acids long. Because the fully tryptic peptides were the substrate of our activity assay, decreased abundance of the peptide indicates higher PK activity. To identify significantly changing peptides based on moderated *t*-tests using the R package protti^79^ followed by a Benjamini–Hochberg correction^80^, we compared peptide abundances at high temperatures (68.6 °C and 72.5 °C) and low temperatures (37 °C and 40.5 °C) for the temperature assay and control and osmolyte (1 M concentration, except for trehalose at 0.5 M) for the osmolyte comparison. The peptides were considered significantly changed if the adjusted *P* value was <0.05 and the | log_2_ (fold change) | value was >1.

We observed significant changes (adjusted *P* value of <0.05, | log_2_ (fold change) | value of >1) in only 6.6% of peptides between the different temperatures applied in our thermal profiling experiment (Supplementary Fig. 1c) and in less than 3.5% of peptides across our tested osmolytes (Supplementary Fig. 1d). To test whether these minor changes in PK activity could influence the thermal profiling readout, we performed independent thermal denaturation experiments using multiple PK concentrations to mimic different PK activities. We observed that rescaling each peptide intensity between 0 and 1 reduced the difference between samples with different PK concentrations while not affecting the expected unfolding effects due to temperature differences.

### Learning peptide intensity profiles across temperatures

We extended the LiP–MS thermal profile analysis beyond the analysis of only thermal unfolding by fitting peptide intensity data along the temperature gradient using a nonparametric Gaussian model instead of the thermodynamic model we had previously used. This extended our analysis beyond peptides that follow a typical sigmoidal unfolding profile. To learn the temperature profiles for each peptide under different conditions, we used Gaussian processes (GP)^81^, which provide a probabilistic framework to learn a nonparametric relationship of peptide abundance to temperature. In detail, we used gpytorch^82^ version 1.4.2 with an ExactGP model choosing a constant mean function, a squared exponential kernel and a Gaussian likelihood. For each peptide, separate GP models for peptide intensities in the absence (control condition) and presence of an osmolyte (osmolyte condition) and a joint model were defined, and model hyperparameters were found by maximizing the sum marginal log likelihood across all models using Adam optimizer with a learning rate of 0.1 and 1,000 iterations. Based on the resulting posterior of the fit, predicted mean abundance profiles and confidence intervals based on 2 s.d. around the mean were found for each peptide and condition. The residual sum of squares between the observed peptide intensities and the predicted intensities were calculated for each peptide and condition to assess the goodness of the fit.

### Clustering of profiles

Fitted values (for 20 temperatures with 2 °C increases) from the learned temperature profiles in the control model were taken for all peptides and clustered into 20 clusters using fuzzy *k* means clustering (FKM.ent function) from the fclust R package^83^, setting the fuzzy degree of entropy parameter to 2. After clustering, the median profiles of the clusters were grouped into three groups. First, we separated the clusters based on whether the median profile showed nonmonotonous behavior (orange group; the difference in peptide intensity between the lowest and highest temperature < 0.5). Next, monotonous clusters were grouped into increasing group (purple; peptide intensity at lowest temperature < peptide intensity at highest temperature) and decreasing group (green; peptide intensity at lowest temperature > peptide intensity at highest temperature). To identify whether the peptide belongs to the same cluster shape following osmolyte addition, clustering was repeated by including fitted values from the models for the control and osmolyte conditions. Peptides were clustered and grouped into three groups as before. For each peptide, probabilities of belonging to individual clusters from the same profile group were summed. A peptide was considered as changing when the difference in probability for a certain main group for control or osmolyte was larger than 0.5.

### Calculation of peptide- and protein-level scores

After fitting of the GP models, distances between the learnt control and osmolyte curves were calculated for the temperature regions where their confidence intervals do not overlap. In the case of overlapping confidence intervals, the distance was set to 0. To study protein stabilization specifically, temperature intervals with intensity changes were classified as binding (changes at the start of the temperature gradient), stabilization (changes at the middle of the temperature gradient) or aggregation (changes at the end of the gradient). The distances between the curves in temperature intervals were summed as a proxy for the area between the curves. To identify whether the change represents protein stabilization or destabilization, clustering was used as described before to identify whether the peptide has a decreasing or increasing profile, and the sign of the area was corrected accordingly. If two areas of stabilization were identified, only the absolute maximal area was considered the peptide-level stabilization score. To summarize the peptide-level stabilization data into protein-level stabilization data, amino acid-level stabilization was calculated to correct for overlapping peptides and peptides of different lengths. To calculate the amino acid-level score, we first calculated the mean value of all peptide-level scores of overlapping peptides at a specific amino acid position to determine whether that position was generally stabilized (mean > 0) or destabilized (mean < 0). For a ‘stabilized position’, a weighted 0.75 quantile of the peptide-level score for all overlapping peptides at a specific position was calculated, weighted by the goodness of fit (summed for both conditions (control and osmolyte)). For a ‘destabilized position’, a weighted 0.25 quantile was calculated. The mean goodness of fit for each position was also calculated. To summarize the information into a single protein-level stabilization score, we first calculated a mean value of all amino acid-level scores to determine whether the protein was generally stabilized (mean > 0) or destabilized (mean < 0). For stabilized proteins, we calculated the weighted 0.75 quantiles for all positions, weighted by the mean goodness of fit. For destabilized proteins, we calculated the weighted 0.25 quantiles for all positions, weighted by the mean goodness of fit. The 0.75 quantile was chosen to maximize the agreement between TPP and LiP–MS data, while keeping the false-positive rate of analysis below 5%. More details about the analysis can be found in the Rmarkdown script (Supplementary Note). To assess the peptide-level FDR of the proposed approach of identifying stabilized proteins from melting curves, the control experiment (that is, no osmolyte) was performed with four replicates that were grouped into two groups and treated as separate conditions. In such a setup, we would expect no significant changes between groups. We observed that the peptide-level FDR was lower than 0.05. For calculation of the protein-level FDR, different quantiles for summarization were considered, ranging from 0.5 to 1 for stabilized protein and from 0.5 to 0 for destabilized protein. A protein was considered significantly changing when the protein-level score was not equal to 0 (Supplementary Fig. 2c). We observed a protein-level FDR of 0.05 when 0.75 quantile (0.25 quantile for destabilized proteins) was used for summarization.

### Comparison of stabilization strength for known binders

Proteins were classified as known binders of glucose, betaine or proline based on UniProt annotations. Analysis was performed on peptide-level stabilization scores, as only few known binders were identified in the literature. Within each dataset, only peptides with significant stabilization scores were considered, and the peptides were classified as originating from a binding protein or not (binding and not binding). Stabilization scores were scaled within a dataset using the following formula to adjust for overall different strengths of different osmolytes, for example, glucose stabilizing the proteome better than glucose:

$$\begin{array}{l}{{\mathrm{Scaled}}}\,{{\mathrm{score}}}\\=\displaystyle\frac{{{\mathrm{Peptide}}}\,{{\mathrm{stabilization}}}\,{{\mathrm{score}}}-{{\mathrm{median}}}\,({{\mathrm{stabilization}}}\,{{\mathrm{scores}}})}{0.7\,{{\mathrm{quantile}}}\,\left({{\mathrm{stabilization}}}\,{{\mathrm{scores}}}\right)-0.25\,{{\mathrm{quantile}}}\,({{\mathrm{stabilization}}}\,{{\mathrm{scores}}})}\end{array}$$

The stabilization scores are all stabilization scores of stabilized peptides of a given osmolyte dataset. Scaled stabilization scores for known binders and nonbinding proteins from glucose, betaine and proline datasets were then combined to produce Fig. 4a. To produce Supplementary Fig. 4a, the same approach was applied, only the binding and nonbinding proteins were mismatched, for example, glucose binding protein was assigned as betaine binding protein to assess whether the subset of binding proteins was generally stabilized better under all osmolyte conditions.

### LiP–MS binding analysis

To study changes of native proteins after small-molecule addition, we focused only on the first two temperatures (37 °C and 40.5 °C) where the majority of the proteins should not be unfolded. Peptide differential abundance was calculated based on *t*-tests, followed by Benjamini–Hochberg multiple testing correction^80^. Because this analysis was performed on nonscaled data (compared to scaled data used for the analysis of thermal stabilization), a more stringent cutoff was used for significance analysis to account for potential changes caused solely due to changes in PK activity that scaling can correct for. The significance cutoff was set to an adjusted *P* value of <0.01 and a | log_2_ (fold change) | value of >1.5. Proteins were identified as significantly changing as soon as one proteotypic significantly changing peptide from that protein was identified.

### Calculation of protein characteristics

We assessed 54 protein physicochemical and biochemical characteristics based on protein sequences (for example, charge, pI and hydrophobicity) on AlphaFold-predicted protein structures^84^ (for example, RASA, secondary structure and disorder) or using a variety of additional sequence-based predictors. Protein characteristics based on protein sequence (protein pI, charge, protein length, hydrophobicity, aliphatic index, hydrophobic moment and percentages of individual amino acids or amino acid groups) were calculated using the Peptides package (https://journal.r-project.org/archive/2015/RJ-2015-001/RJ-2015-001.pdf) in R. The predictions SCRIBER score^85^, DRNApredDNAscore^86^, DRNApredRNAscore^86^, MoRFchibi^54^, DFLpred, DisoRNAscore, DisoPROscore and DisoDNAscore were downloaded from DescribeProt^87^. Solubility (CamSol) was calculated using CamSol software^88^. Aggregation prediction was calculated using TANGO^89^. Amino acid-level information was summarized by calculating the percentage of amino acids in the aggregation-prone region (percentage_agg) or the longest consecutive stretch of aggregation-prone amino acids (longest_agg). RASAs and secondary structures were calculated using AlphaFold-predicted structures^84^ with the Bio.PDB package in Python using the DSSP module with default settings. Individual values for RASA were calculated by calculating the average RASA for the whole protein. Individual values for each structural element (helix, sheet or coil) were calculated as a percentage of amino acids of a certain structure to the whole protein. The radius of gyration was calculated using AlphaFold structures with the Bio.PDB package (Rg function) with default settings. To identify whether two groups (stabilized versus nonstabilized, high correlation versus low correlation and increased aggregation versus rest) of proteins significantly differ in certain protein characteristics, *t*-tests were performed, followed by Benjamini–Hochberg corrections^80^. The difference between mean values for the two groups was calculated, and the control value was always subtracted from the test group to calculate whether the characteristics were higher or lower in the test group.

### Identification of nonprecipitators

Proteins were characterized as precipitators or nonprecipitators based on the TPP dataset^47^. Proteins were considered nonprecipitators if at the highest temperature more than 50% of the protein, relative to 37 °C, remained in the soluble fraction.

### Analysis of differential scanning fluorimetry data

Raw fluorescence intensities were scaled between 0 and 1 using the following formula: *x*′ = [*x* – min(*x*)]/[max(*x*) – min(*x*)]. Scaled intensities as a function of temperature were fit to the following equation: *S* = ((*S*_f_ + *m*_f_ × *T*) + (*S*_u_ + *m*_u_ × *T*) × e[(∆*H*_m_/*RT*) × ((*T* – *T*_m_)/*T*_m_)])/(1 + e[(∆*H*_m_/*RT*) × ((*T* – *T*_m_)/*T*_m_)]). Here, *S* is the scaled fluorescence signal in arbitrary units, *S*_f_ and *S*_u_ are the scaled fluorescence signals of the folded and unfolded states at 0 K, respectively, in arbitrary units, *m*_f_ and *m*_u_ are the linear dependencies of *S*_f_ and *S*_u_, respectively, on temperature (that is, slopes of the pre- and post-transition baseline, respectively) in arbitrary units K^−1^, *T* is the temperature in K, *T*_m_ is the melting temperature in K, ∆*H*_m_ is the unfolding enthalpy at the *T*_m_ in J mol^−1^, and *R* is the gas constant, 8.314 J K^−1^ mol^−1^. The fitting was performed as previously described^42^.

### Analysis of thermal protein profiling data

Protein-level quantification data were extracted from Spectronaut without data normalization. Protein abundance relative to 37 °C was calculated to determine the amount of protein in the soluble fraction. Scaled abundances were processed, fit and analyzed the same way as peptide-level data from the LiP–MS study without summarization.

### Spearman correlation analysis

To calculate whether individual proteins are stabilized in a similar manner as the whole lysate, the Spearman correlation between protein stabilization score and mean stabilization score was calculated for all proteins reproducibly measured under all conditions. To obtain a random distribution, we calculated the Spearman correlation between protein stabilization score and randomized mean stabilization score shuffled between different osmolyte conditions independently for each protein. The calculation of randomized correlations was repeated 1,000 times to assess the variability of random distribution.

### Identification of proteins with changed aggregation abundance

To identify the proteins where protein precipitation is affected, we focused on the last three temperatures (68.8, 72.5 and 76 °C) of TPP measurements. To identify whether the protein precipitates significantly more or less after the addition of osmolyte, *t*-tests were performed on scaled values, followed by Benjamini–Hochberg multiple testing correction^80^. The significance cutoff was set to an adjusted *P* value of <0.05 and | log_2_ (fold change) | value of >1. To identify whether LiP–MS shows significant changes in protein aggregation/structure of protein aggregates, we focused on the last two temperatures (72.5 and 76 °C). We performed significance analysis, as described earlier, at the peptide level. Proteins were considered significantly changing as soon as a single significantly changing peptide was identified.

### Gene ontology enrichment analysis

We tested the proteins where TMAO promotes protein aggregation as defined above (Identification of proteins with changed aggregation) for functional enrichments using the topGO package in R^90^. We downloaded current annotation files for *E. coli* (http://current.geneontology.org/annotations/ecocyc.gaf.gzm, accessed 2 June 2020). To focus on the most informative terms, we tested for enrichment with Fisher’s exact tests using the elim-algorithm in topGO^90^. We performed the test for biological processes, cellular compartments and molecular functions. Only terms with adjusted *P* values of <0.01 after the Benjamini–Hochberg *P* value correction^80^ were considered; however, *P* values and unadjusted *P* values are displayed.

### Domain-level analysis

Peptides were mapped to domains based on Pfam annotation^91^. A peptide was considered part of a domain when a single amino acid of a peptide was part of the domain. Proteins with at least two domains with at least three peptides mapping to each of the domains were considered for analysis. Differential analysis using *t*-tests (two domains) or analysis of variance (more than two domains) was performed to identify proteins with domains with significantly different stabilization.

### Data visualization and processing

Data visualization was performed using R version 4.0.5 and the ggplot2 (ref. ^92^) and ComplexHeatmap^93^ packages for plotting.

### Reporting summary

Further information on research design is available in the Nature Portfolio Reporting Summary linked to this article.

## Online content

Any methods, additional references, Nature Portfolio reporting summaries, source data, extended data, supplementary information, acknowledgements, peer review information; details of author contributions and competing interests; and statements of data and code availability are available at 10.1038/s41589-024-01568-7.

### Supplementary information

Supplementary Information Supplementary Figs. 1–6 and Note.

Reporting Summary

Supplementary Data 1 Analysis of thermal stabilization by osmolytes.

Supplementary Data 2 Binding analysis following the addition of osmolytes.

Supplementary Data 3 Analysis of osmolyte effects on protein aggregation.

Supplementary Data 4 Analysis of osmolyte effects on the human proteome.

## Data Availability

All MS proteomics data have been deposited at ProteomeXchange Consortium via the PRIDE partner repository with the dataset identifier PXD036186. UniProt fasta databases for *E. coli* (strain K12, organism ID 83333) were accessed in November 2020 via the UniProt databases download page (https://www.uniprot.org/downloads). Protein structures for DnaK (PDB ID 4JNE) were downloaded in 2022 from the Protein Data Bank website (https://www.rcsb.org/pdb). AlphaFold predictions for the whole *E. coli* proteome were downloaded in August 2021. The DescribeProt database for the whole *E. coli* proteome (http://biomine.cs.vcu.edu/servers/DESCRIBEPROT/download.html) was downloaded in February 2021. The TPP dataset from a previously published study^47^ (dataset EV3 of the publication) was downloaded in 2020. All other data needed to evaluate the conclusions described in the paper are present in the Supplementary Information.

## Code availability

The custom R script for data analysis is available via GitHub at https://github.com/PicottiGroup/Thermal_unfolding, together with Rmarkdown showing how to perform the analysis, and on Zenodo (10.5281/zenodo.10514118).
