# Supplementary material for: In situ analysis of osmolyte mechanisms of proteome thermal stabilization
Source: Nat Chem Biol. 2024 Feb 29;20(8):1053–65. doi: 10.1038/s41589-024-01568-7 (PMC11288892; doi:10.1038/s41589-024-01568-7)
Supplement: Supplementary file 1 — Supplementary Figs. 1–6 and Note. [file 41589_2024_1568_MOESM1_ESM.pdf]

nature chemical biology

Article

https://doi.org/10.1038/s41589-024-01568-7

In situ analysis of osmolyte mechanisms of proteome thermal stabilization

In the format provided by the authors and unedited

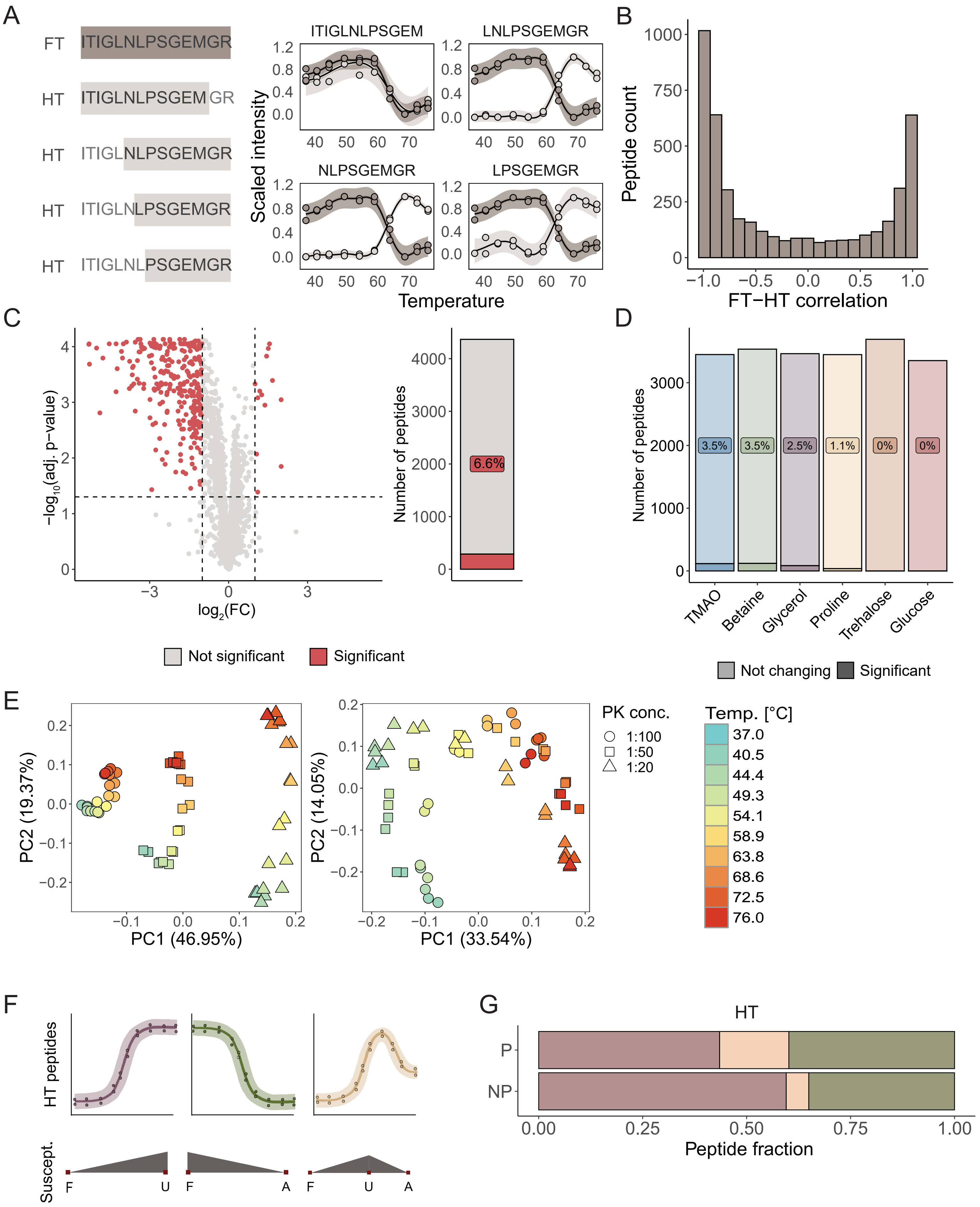
A
FT ITIGLNLPSGEMGR
HT ITIGLNLPSGEMGR
HT ITIGLNLPSGEMGR
HT ITIGLNLPSGEMGR
HT ITIGLNLPSGEMGR
ITIGLNLPSGEM
LNLPSGEMGR
NLPSGEMGR
LPSGEMGR
Scaled intensity
Temperature
B
Peptide count
FT−HT correlation
C
−log10(adj. p−value)
log2(FC)
6.6%
Number of peptides
Not significant
Significant
D
Number of peptides
3.5%
3.5%
2.5%
1.1%
0%
0%
TMAO
Betaine
Glycerol
Proline
Trehalose
Glucose
Not changing
Significant
E
PC2 (19.37%)
PC1 (46.95%)
PC2 (14.05%)
PC1 (33.54%)
PK conc.
1:100
1:50
1:20
Temp. [°C]
37.0
40.5
44.4
49.3
54.1
58.9
63.8
68.6
72.5
76.0
F
HT peptides
Suscept.
F
U
F
A
F
U
A
G
HT
P
NP
Peptide fraction

Supplementary Fig. 1: LiP-MS thermal profiling to study osmolyte effects. (A) Example sequence of a fully tryptic peptide (FT, dark grey) with overlapping half tryptic peptides (HT, light grey) and their corresponding LiP-MS thermal profiles. Note that while profiles are not identical, FT and HT peptides all report the same melting temperature. Shaded area represents confidence interval of the fit. (B) Distribution of Pearson correlation coefficients calculated between the thermal profile of pairs of FT and HT peptides. (C) Differential analysis for a control peptide sample digested with proteinase K (PK) at high temperatures (68.2°C, 72.5°C) vs low temperatures (37°C, 40.5°C). Dashed lines indicate significance cutoff (adj. p-value <0.05, |log2FC| > 1). The bar plot (right) shows the number of all peptides analysed (grey) vs significantly changing peptides (red); the percentage of significantly changing peptides is indicated. (D) Differential analysis for a control peptide sample digested with proteinase K (PK) in the presence or absence of the indicated osmolytes. The bar plot shows the number of all peptides analysed (light colours) vs significantly changing peptides (dark colours) (adj. p-value <0.05, |log2FC| > 1); the percentage of significantly changing peptides is indicated. (E) Principal component analysis of LiP-MS thermal denaturation experiment performed at three different PK concentrations before (left) and after (right) applied scaling. Each point represents a measured sample. After scaling, the difference between PK concentrations disappears while temperature differences remain. (F) Interpretation of HT peptide behaviour in the three cluster groups from 1C. We interpret changing HT peptide intensity as a function of increased or decreased proteolytic susceptibility (Suscept.), as indicating that protein is in a folded (F), unfolded (U) or aggregated (A) state. For FT peptides, the opposite effect (i.e., a flipped thermal profile) is expected (Figure 1D). (G) Percentage of cluster groups from Fig. 1C separated by proteins defined as non-precipitators (NP) and precipitators (P) in thermal protein profiling; only HT peptides are shown.

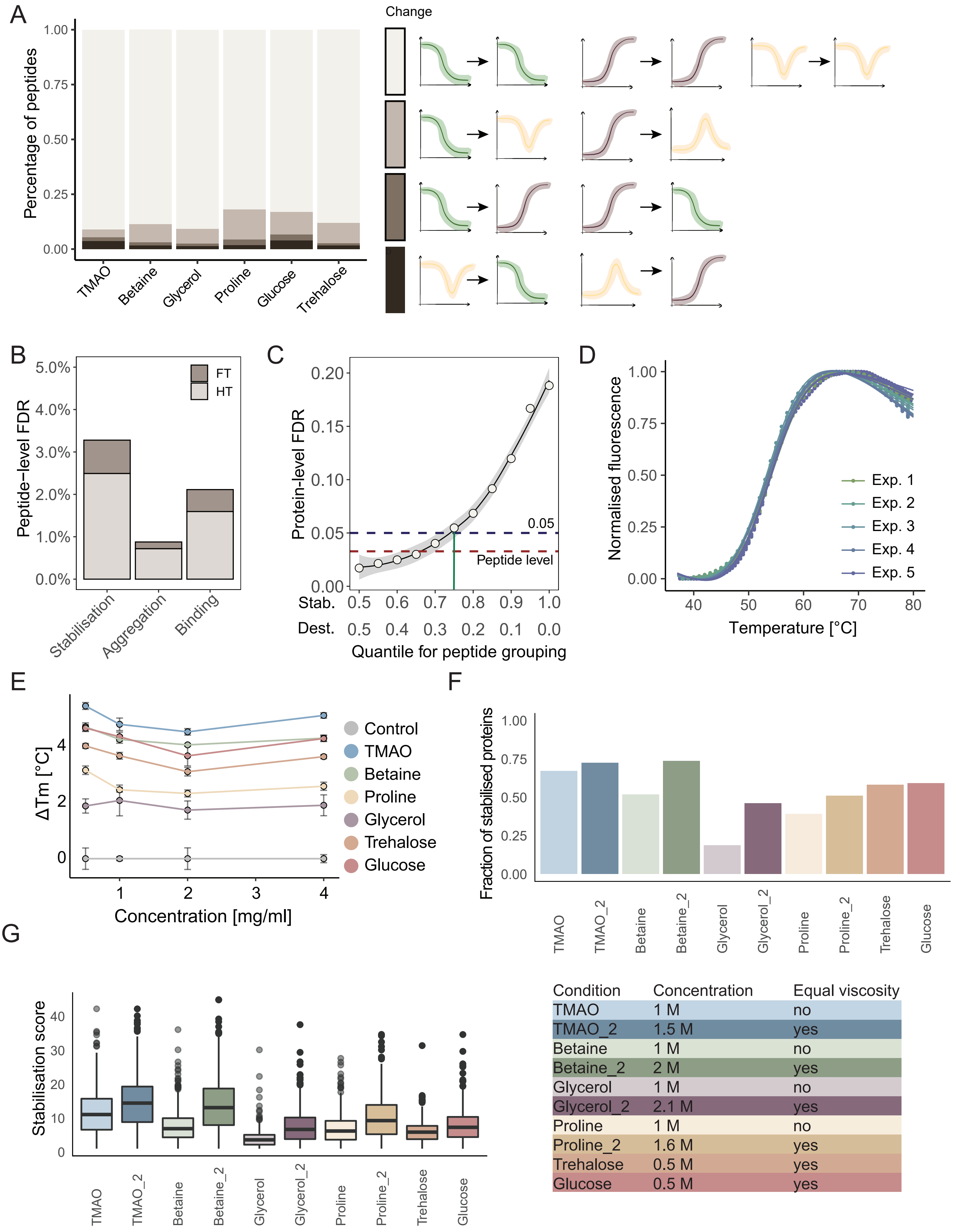

Condition	Concentration	Equal viscosity
TMAO	1 M	no
TMAO_2	1.5 M	yes
Betaine	1 M	no
Betaine_2	2 M	yes
Glycerol	1 M	no
Glycerol_2	2.1 M	yes
Proline	1 M	no
Proline_2	1.6 M	yes
Trehalose	0.5 M	yes
Glucose	0.5 M	yes

Supplementary Fig. 2: Osmolytes have a global effect on protein stability. (A) The plots (right) show peptide profile changes upon osmolyte addition. The plot (left) shows the fractions of peptides that correspond to these changes, coloured as on the right. (B) Peptide-level false discovery rate (FDR; see Methods) is shown for indicated analyses. (C) Protein-level FDR calculated for different quantiles used in summarisation of peptide-level scores into a protein-level score. The x-axis shows quantiles used for summarisation of stabilised (Stab.) or destabilised (Destab.) proteins. FDR is calculated by combining significantly stabilised or destabilised proteins. The red line represents peptide level FDR and blue line represents FDR level of 0.05, corresponding roughly to a 0.75:0.25 quantile split for stabilised:destabilised proteins (green line). Shaded area represents confidence interval of the fit. (D) Melting curves for E. coli lysate measured by Differential Scanning Fluorimetry at protein concentration of 0.5 mg/ml (Experiment 1), 1 mg/ml (Experiments 2, 3) and 2 mg/ml (Experiments 4, 5). Two E. Coli lysates were used (Lysate 1: Experiments 1, 2, 4, Lysate 2: Experiments 3, 5). (E) The plot shows the difference in lysate melting temperature (ΔTm) between osmolyte and control conditions for different E. coli lysate concentrations; ΔTm is relative to the mean Tm in control at each concentration. Points represent the mean +/- standard deviation from a single experiment (n=5 technical replicates). (F) Fraction of stabilised proteins out of all detected proteins, for each osmolyte at equalised molarity (light colours) or viscosity (darker colours). The table (bottom) shows osmolyte concentrations used to match the viscosity of 1M glucose and 0.5 M trehalose. (G) Distribution of stabilisation scores for proteins significantly stabilised by each osmolyte. Osmolyte concentrations as in F. Horizontal lines define the median and boxes the 25th and 75th percentiles; whiskers represent maximum and minimum values. Boxplots show stabilisation scores calculated based on a single experiment conducted over 10 temperatures (2 LiP replicates each). Each plot represents at least 200 proteins.

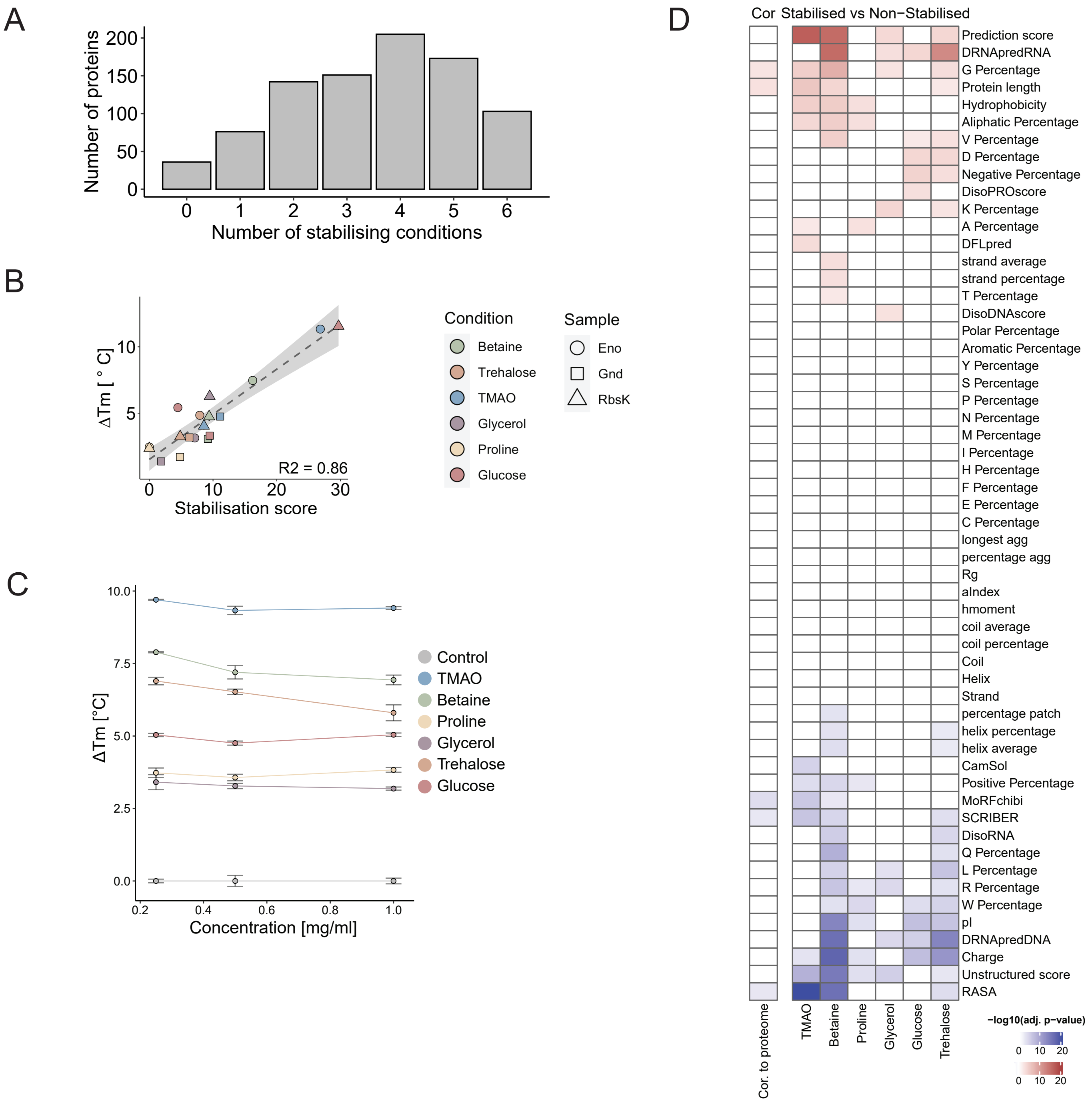

Supplementary Fig. 3: Biophysical features of osmolyte-stabilised proteins. (A) The number of proteins stabilised by different numbers of osmolytes is plotted. Zero indicates proteins not stabilised by any tested osmolyte. (B) Linear regression between DSF-calculated ΔTm and protein stabilisation score for combined data from Figure 3D. Shaded area represents the confidence interval of the linear fit (dashed line). (C) The plot shows the difference in enolase melting temperature (ΔTm) between osmolyte and control conditions, at different concentrations of enolase. ΔTm is calculated relative to the mean melting temperature of the control condition at each protein concentration. Results are based on a single experiment (n = 4 technical replicates). Points represent the mean +/- standard deviation. (D) The heatmap shows the significantly different biophysical features between proteins with high vs low correlation of the stabilisation score with that of the global proteome (first column, Cor) or for proteins significantly stabilised vs not stabilised by individual osmolytes. The colour intensity indicates significance levels. The colour indicates whether the feature is higher (red) or lower (blue) in proteins with high correlation (first column) and in stabilised proteins (other columns) compared to the rest. Feature significance was determined by two-sided t-test followed by a correction for multiple hypothesis testing with the Benjamini-Hochberg method. See Methods for how individual features were measured or predicted.

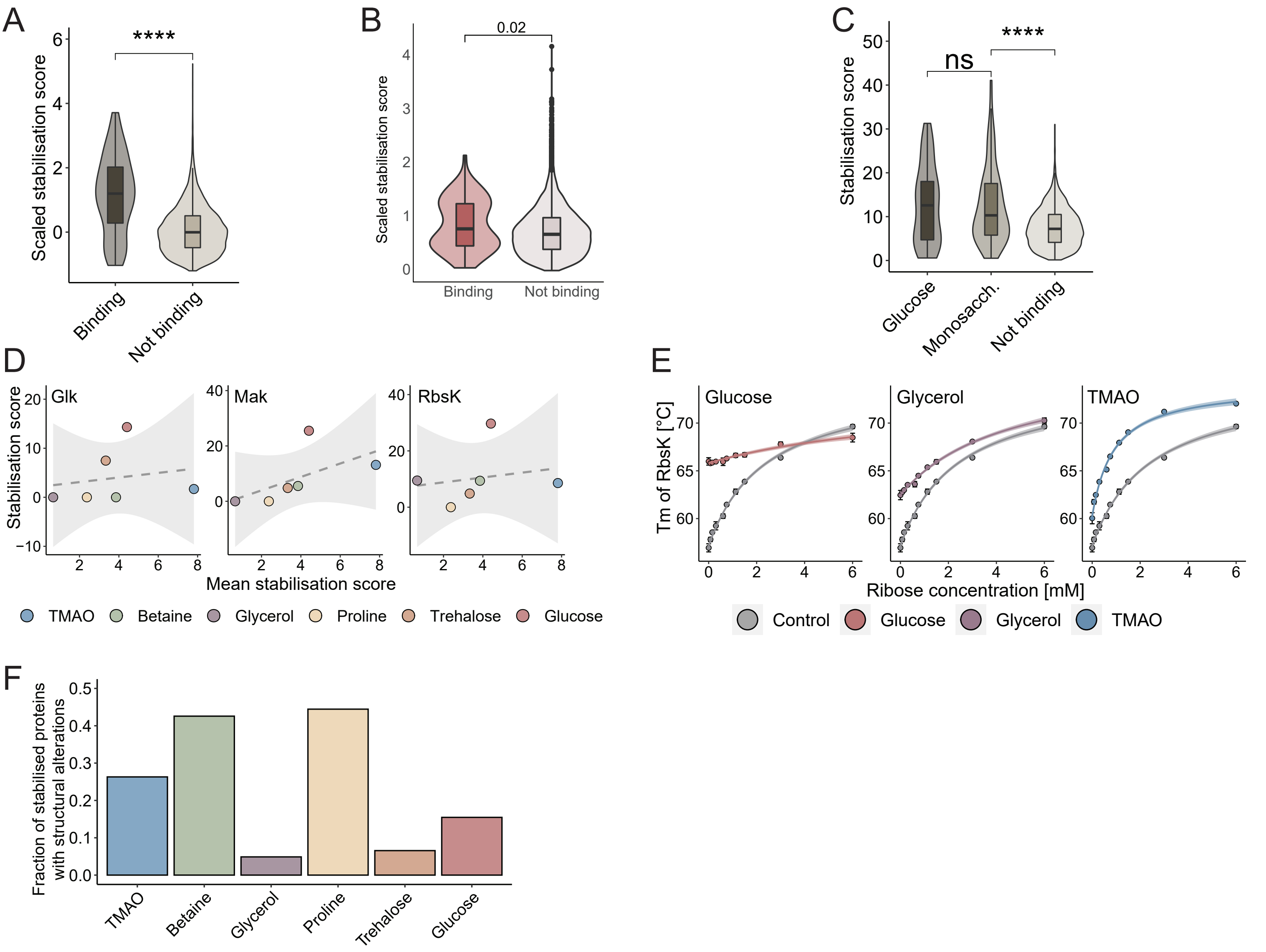

Supplementary Fig. 4: Direct binding of osmolytes and effects on native protein structure. (A) Distributions of scaled peptide stability scores for proteins stabilised by proline, betaine or glucose, plotted for known binders of each of these osmolytes (dark grey) and for all stabilised proteins (grey). Scores were derived from a single experiment, at 10 temperatures (2 LiP replicates each; n= 94 peptides from 13 proteins (binding); 12114 peptides from 1069 proteins (not binding)). Horizontal lines define the median and boxes the 25th and 75th percentiles; whiskers represent the maximum and minimum values. Significance is determined using two-sided Wilcoxon test (**** p-value < 0.0001). (B) As in A but plotted for all stabilised proteins (grey) and for known binders of mismatched osmolytes (red) (e.g, known betaine-binders from the proline and glucose datasets). Plots and statistical analysis as in A. (C) Distributions of peptide-level stabilisation scores for proteins stabilised by glucose, plotted for known binders of glucose (dark grey), of monosaccharides (grey), and for all stabilised proteins (light grey, right). Plots and statistical analysis as in A. (n= 48 peptides from 9 proteins (glucose), 63 peptides from 13 proteins (monosaccharides), 4619 peptides from 854 proteins (not binding)). (D) Linear regression between the stabilisation score for Glucokinase (Glk), Fructokinase (Mak) and Ribokinase (RbsK) in the presence of different osmolytes, and the mean stabilisation score across all detected proteins. Each point represents one osmolyte condition. Shaded area represents the confidence interval of the linear fit (dashed line). (E) DSF-measured melting temperature of Ribokinase (RbsK) at varying ribose concentrations in control (grey line) and upon addition of 1 M glucose (red), 1 M glycerol (purple) or 1 M TMAO (blue). Error bars show mean +/- standard deviation (n=5 replicates). Shaded area represents confidence interval of the fit. (F) The fraction of stabilised proteins that also show a change in native structure for each osmolyte, as determined by LiP-MS.

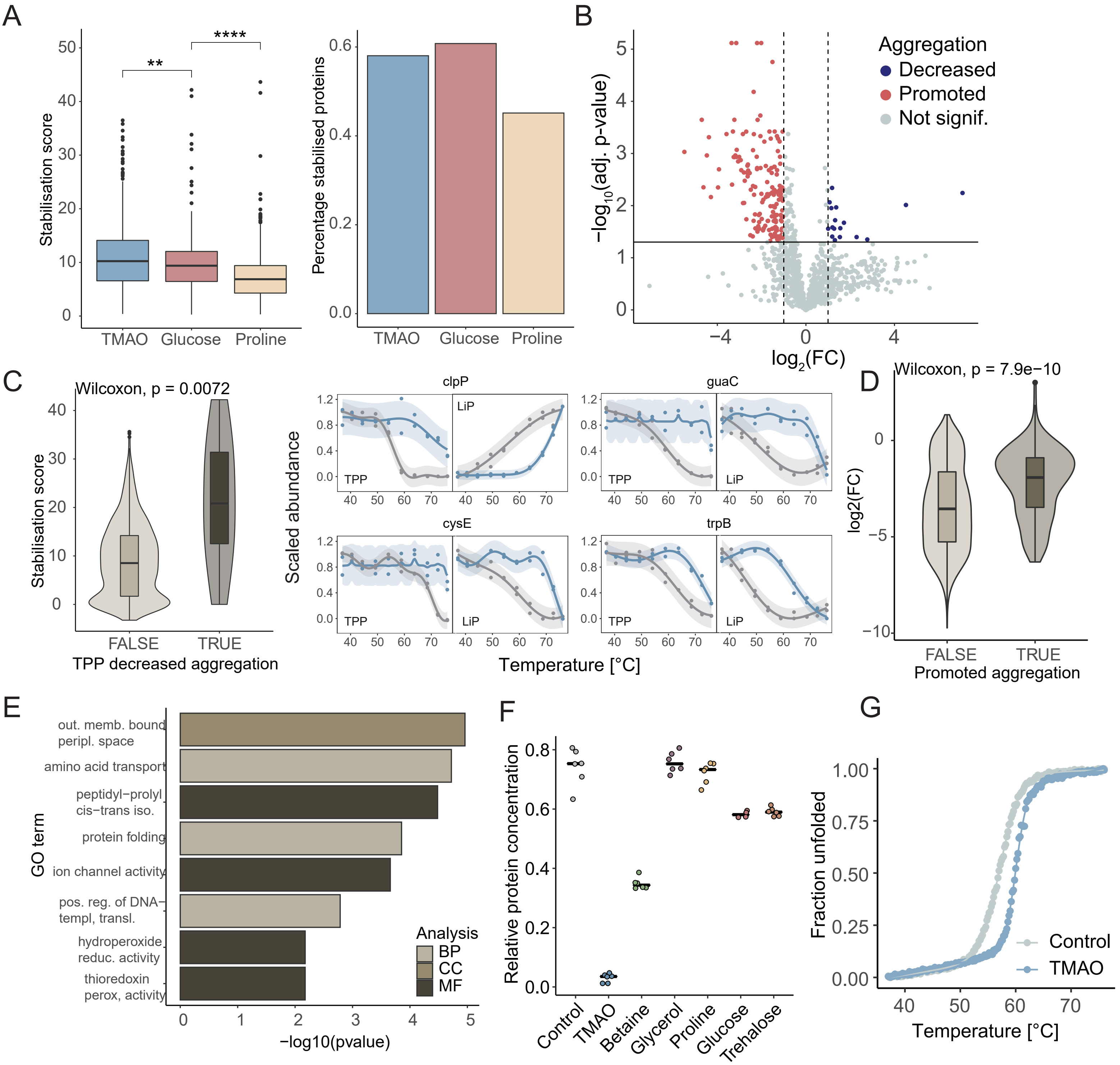

Supplementary Fig. 5: Osmolyte effects on protein aggregation in native lysates. (A) TPP-measured fraction of stabilised proteins out of all detected proteins in the indicated osmolyte condition (right) and distribution of stabilisation scores for the corresponding stabilised proteins (left). Horizontal lines define the median and boxes the 25th and 75th percentiles; whiskers represent the maximum and minimum values. Significance is determined using two-sided Wilcoxon test (** p-value < 0.01, **** p-value < 0.0001). Scores were derived from a single experiment at 10 temperatures (2 technical replicates). Each plot represents all stabilised proteins per condition (n= 549 proteins (TMAO), 626 proteins (glucose), 353 proteins (proline)). (B) Differential analysis of protein abundance in soluble fraction between control and 1M TMAO conditions, at 68.2°C, 72.5°C and 76°C. Dashed lines indicate significance cutoff (adj. p-value <0.05, |log2FC| > 1). Each dot represents an individual protein. Proteins with significantly promoted (red) or decreased (blue) aggregation are indicated. (C) Distribution of LiP-MS-calculated stabilisation score for proteins with decreased aggregation in TMAO (TRUE, 8 proteins with >1 peptide per protein) compared to the rest (FALSE, 686 proteins with > 1 peptide per protein). Example TPP (protein) and LiP-MS (peptide) profiles for selected proteins under control (grey) and TMAO (blue) conditions are shown (right). Shaded area represents confidence interval of the fit. Plots and statistics as in A. (D) Distribution of fold changes (FC) between 76°C and 37°C in the absence of added osmolytes, plotted for proteins where TMAO promotes aggregation (TRUE, 158 proteins) vs the rest (FALSE, 1094 proteins)). Higher FC means protein mostly remains in soluble fraction even at higher temperatures. Plots and statistics as in A, C. (E) GO enrichment analysis for proteins where TMAO promotes aggregation. BP, biological processes, MF, molecular functions, and CC, cellular compartments. Significantly enriched terms (p-value < 0.01) for all three analyses are shown. (F) Relative concentration of soluble ribosomal recycling factor (Frr) after heating (70 °C) of the purified protein under indicated conditions, scaled for initial protein concentration. (n=6 replicates). (G) Circular dichroism-measured thermal denaturation profile of Frr in the indicated conditions.

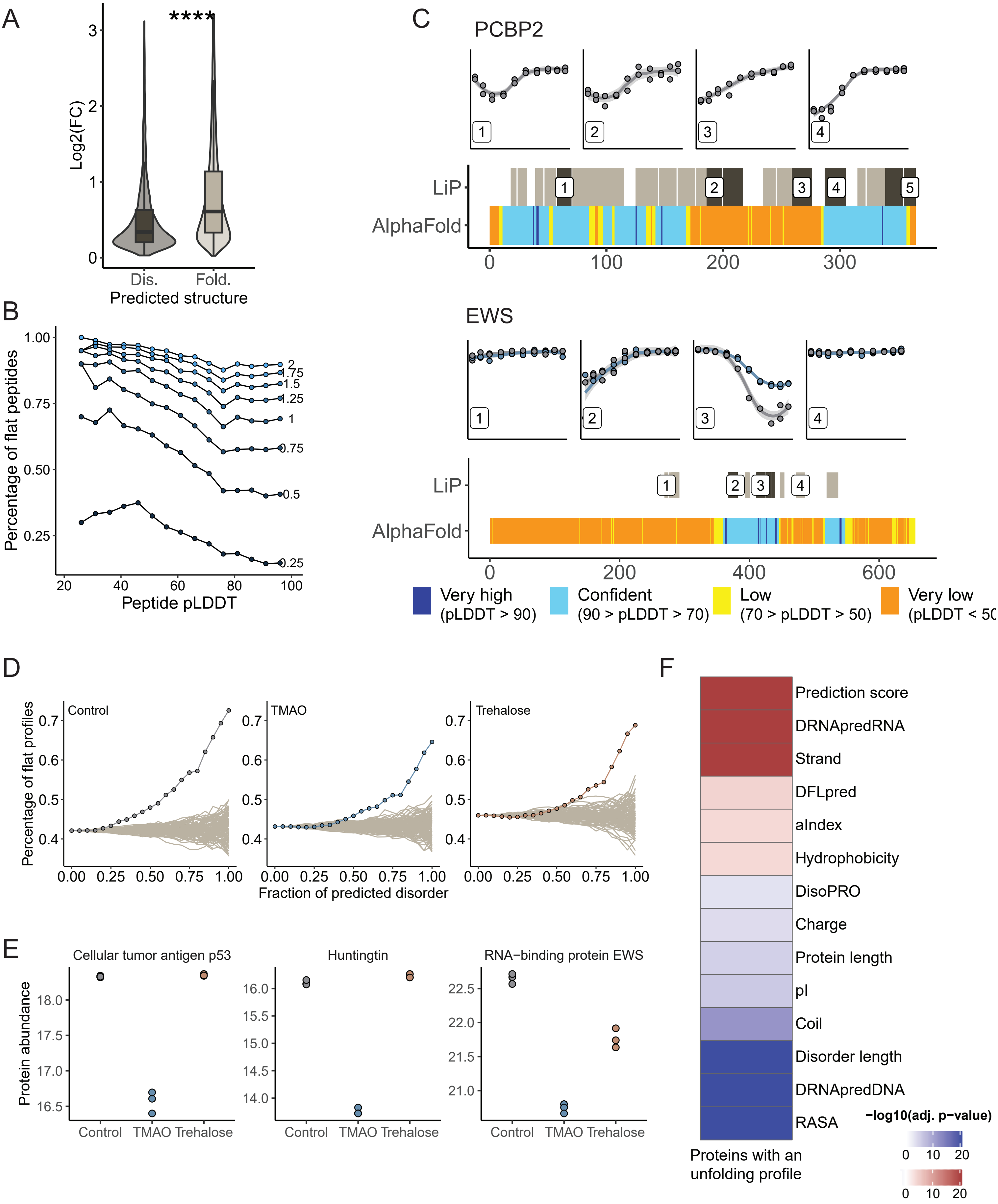
A
Log2(FC)
****
Dis.
Fold.
Predicted structure
B
Percentage of flat peptides
Peptide pLDDT
2
1.75
1.5
1.25
1
0.75
0.5
0.25
C
PCBP2
LiP
AlphaFold
EWS
Very high
(pLDDT > 90)
Confident
(90 > pLDDT > 70)
Low
(70 > pLDDT > 50)
Very low
D
Control
TMAO
Trehalose
Percentage of flat profiles
Fraction of predicted disorder
E
Cellular tumor antigen p53
Huntingtin
RNA−binding protein EWS
Protein abundance
Control
TMAO
Trehalose
F
Prediction score
DRNApredRNA
Strand
DFLpred
aIndex
Hydrophobicity
DisoPRO
Charge
Protein length
pI
Coil
Disorder length
DRNApredDNA
RASA
Proteins with an unfolding profile
−log10(adj. p−value)

Supplementary Fig. 6: Characterisation of osmolyte effects on human proteome. (A) Comparison of thermal profile flatness for peptides with low predicted disorder (light grey, Fold., 44701 peptides) and high predicted disorder (dark grey, Dis., 2794 peptides) from a single experiment at 10 temperatures (2 LiP replicates). The difference between the maximum and minimum scaled peptide intensity in the thermal profile is plotted in each case. Horizontal lines define the median and boxes the 25th and 75th percentiles; whiskers represent the maximum and minimum values (**** $p < 0.0001$, two-sided Wilcoxon test). (B) The percentage of flat profiles is plotted across the predicted disorder score (AlphaFold pLDDT) for all detected peptides (low score indicates high disorder). The cutoff for profile flatness (log2 difference between min and max intensity across the temperature gradient) is indicated for each curve. (C) Analysis of predicted disorder in regions of PCBP2 (top) and EWS (bottom) with different thermal melting behaviour. The plots (top) show thermal profiles of 4 example peptides mapping to the indicated regions along the protein sequence. For PCBP2, profiles in the control condition are shown; for EWS, the control (grey) and TMAO (blue) conditions are shown. The upper barcode (LiP) for each protein shows all peptides (dark grey) for which we could measure a thermal melting profile (based on goodness of fit to a sigmoidal profile) out of all detected peptides (light grey). The lower barcode shows the pLDDT prediction score, where very low score typically corresponds to disordered protein regions. All barcodes are arranged along the protein sequence. (D) The fraction (percentage) of peptides with flat profiles is plotted (lines with dots) for proteins with increasing fractions of predicted disorder. All detected peptides/proteins are plotted. The grey lines show tests in which the protein disorder was randomised. (E) The plots show protein abundance of p53, huntingtin and EWS in the soluble fraction under the indicated conditions. (F) The heat map shows significantly enriched or depleted features for human proteins that showed thermal unfolding profiles, relative to all detected proteins. Feature significance was determined by two-sided t-test and multiple hypothesis testing (Benjamini-Hochberg).

Supplementary Note

Analysis Pipeline

2023-12-07

Contents

1 Introduction 1

2 Spectronaut to python 2

3 Learning peptide intensity profiles along temperature 3

4 Python to list 4

5 Calculate area: Quantify and classify the significant differences 4

6 Clustering 6

7 Find significant peptides 8

8 Peptide fasta matching 8

9 Aggregate scores 8

10 Running the pipeline 10

10.1 Setup . . . 10
10.2 Spectronaut_to_python() . . . 11
10.3 GP_fitting() . . . 11
10.4 Python_to_list() . . . 12
10.5 Calculate_area() . . . 12
10.6 Clustering() . . . 12
10.7 Find_significant_peptides() . . . 12
10.8 Peptide_fasta_matching() . . . 12
10.9 Aggregate_scores() . . . 12

1 Introduction

LiP-MS based thermal profiling enables proteome-wide analyses of protein thermal stabilisation upon addition of small molecule with a peptide-level resolution. The goal of the approach is to identify significantly stabilised proteins and protein regions and differentiate between the changes observed due to changes in native structure (eg. binding), changes in protein thermal stability (stabilisation or destabilisation) and changes in protein aggregation.

This document provides a starting point for analysing data obtained by LiP-MS based thermal profiling. The different steps in the analysis pipeline are explained to facilitate the reproducibility of the results as well as to allow others to analyze their own data. Below is shown an overview of the whole analysis pipeline. In the following sections, each step is shortly explained and if applicable, examples are shown.

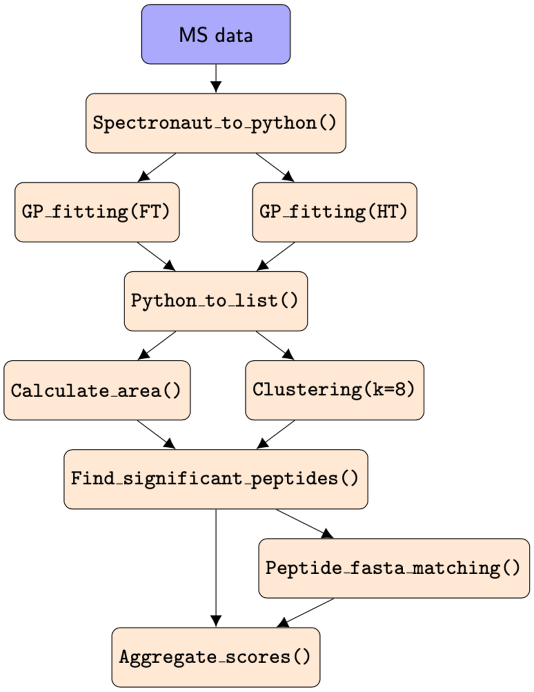

Overview of the data analysis workflow.

2 Spectronaut to python

After acquiring the data on the mass spectrometer in DIA mode, the data was searched using Spectronaut 14 (see method section for details). With the following function Spectronaut_to_python(), this data frame is converted into appropriate format for the next step, where we apply Gaussian process (GP) to learn the temperature profiles for each peptide at different conditions.

The Spectronaut output contains the following columns:

- **R.FileName**: name of raw MS data
- **PG.FastaFiles**: 200333_ecoli
- **PG.ProteinAccessions**: Uniprot identifier
- **PG.ProteinDescription**: Uniprot protein description
- **PG.ProteinNames**: Uniprot protein name
- **PEP.IsProteotypic**: Is the peptide unique to the proteome? (True or False)
- **PEP.StrippedSequence**: Peptide sequence
- **PEP.DigestType...Trypsin.P.**: Is the peptide fully or half tryptic (Specific, Specific-C or Specific-N)
- **PEP.Quantity**: Raw intensity measure of the peptide
- **EG.ModifiedSequence**: Modified peptide sequence
- **Temperature**: Temperature in degree celsius
- **Condition**: Condition (eg. Small molecule or Control)
- **replicate**: Replicate number (1,2 or 3)

The function Spectronaut_to_python takes the previously described data frame and extracts intensities grouped by sequence, temperature and condition. In this step, we also apply the following filters:

- Measurements with raw intensity (*PEP.Quantity*) lower than 100 are removed
- Non-proteotypic peptides are removed
- Contaminants and indexed retention-time peptides (iRT) or in general peptides not originating of the *E. coli* proteome are discarded.
- Peptides containing more than 20% of missing values are removed.

Additionally, the peptide intensities are scaled, using min-max scaling according to the following formula, separately for each peptide and condition.

$$x' = \frac{x - \min(x)}{\max(x) - \min(x)}$$

Finally, Spectronaut_to_python() splits the dataframe into 2 lists according to their trypticity. Both files will contain the following columns:

- **uniqueID**: peptide sequence
- **condition**: Condition (eg. Small molecule or Control)
- **x**: Temperature in degree Celsius
- **y**: Scaled peptide intensity

3 Learning peptide intensity profiles a long temperature

In this step we apply Gaussian processes (GP) to learn the temperature profiles for each peptide in different conditions. The approach was applied to filtered and scaled data from the step a bove. In detail, we used gpytorch version 1.4.2 with an ExactGP model choosing a constant mean function, a squared exponential kernel and a Gaussian likelihood. For each peptide, separate GP models for the peptide intensities in absence (control condition) and presence of an osmolyte (osmolyte condition) as well as a joint model were defined and model hyperparameters were found by maximizing the sum marginal log-likelihood across all models using Adam optimizer with a learning rate of 0.1 and 1000 iterations. Based on the resulting posterior of the fit, predicted mean abundance profiles and confidence intervals based on 2 standard deviations around the mean were found for each peptide and condition. The residual sum of squares between the observed peptide intensities and the predicted intensities are calculated for each peptide and condition to assess the goodness of the fit.

For both fully and half tryptic peptides we obtain two .csv files. One contains the newly fitted points for each peptide (solution_Small_molecule_FT.csv) and the second one contains information about the goodness of fit for each peptide (MLL_Small_molecule_FT.csv).

To highlight the different steps, we take the peptide QAVTNPQNTLFAIK as an example. Panel A below shows the raw MS data. After running the function Spectronaut_to_python(), we have scaled the intensity as shown in panel B. Finally, after performing GP fitting, we obtain fitted curves for each condition as well as for all data points combined as shown in panel C. Additionally, we compute confidence intervals for the fits (in panel D), which are later used to calculate the area between the curves.

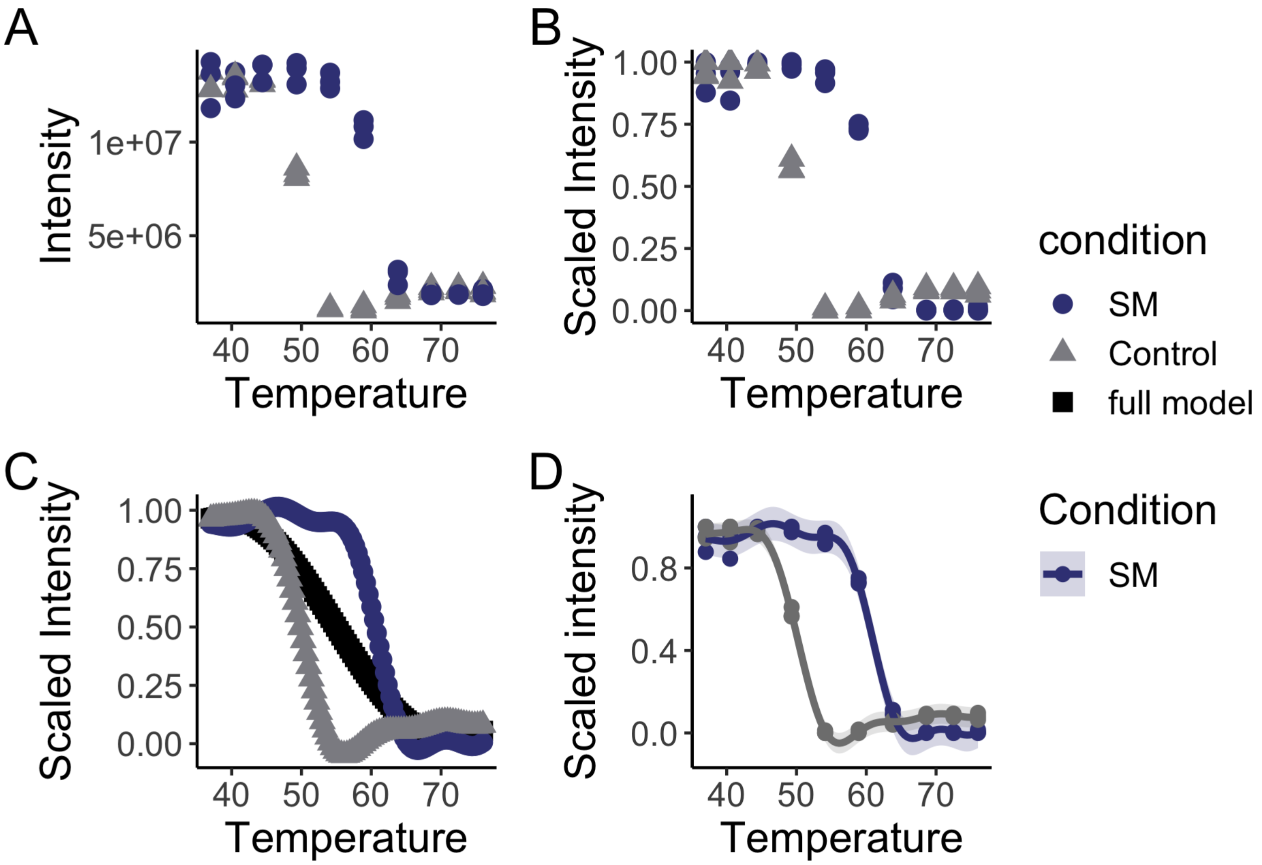

Example output for peptide QAVTNPQNTLFAIK. A) Raw MS data B) Scaled MS data C) GP fitted data D) GP fitted data with confidence intervals. The line represents the GP fit and the shaded area represents the confidence intervals.

4 Python to list

Since the GP modeling was done in python, we merge the output again with the metadata about the run and the peptides such as trypticity and the protein, from which the peptide originated. Furthermore, half and fully tryptic peptides are now merged back into a single dataframe.

5 Calculate area: Quantify and classify the significant differences

After the fitting of the GP models, distances between the learnt control and osmolyte curves were calculated for the temperature regions where their confidence intervals do not overlap. In the case of overlapping confidence intervals, the distance was set to 0. To study specifically protein stabilisation, temperature intervals with intensity changes were classified as binding (changes at the start of the temperature gradient), stabilisation (changes at the middle of the temperature gradient) or aggregation (changes at the end of the gradient). The distances between the curves in temperature intervals were summed up as a proxy for the area between the curves. For each region, an example is shown in the plots below.

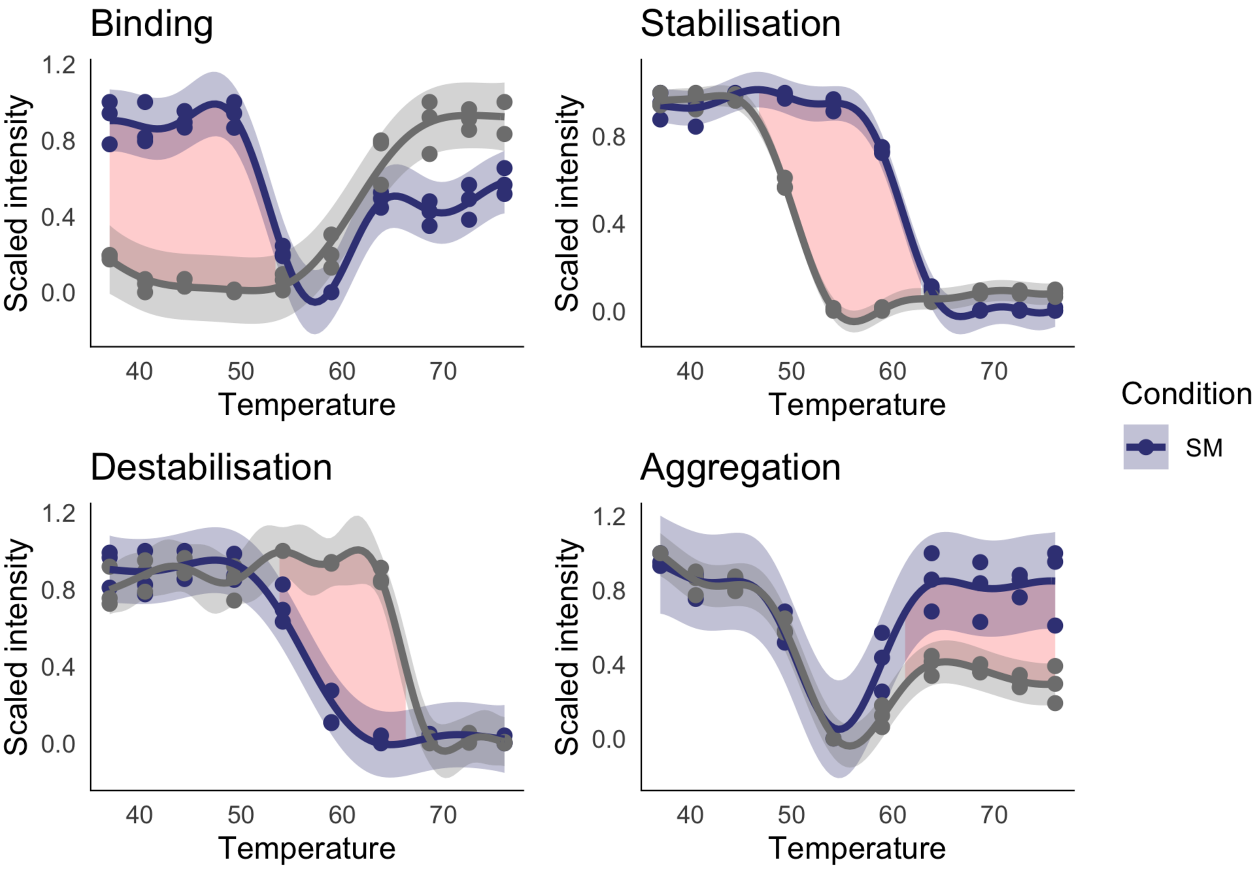

Example peptides showing binding, (de-)stabilisation and aggregation effects. The line represents the fit and the shaded area represents confidence intervals of the fit. The respective areas between the curves, representing binding, (de-)stabilisation and aggregation are highlighted in red.

To illustrate better the calculation of the stabilisation score, we take the following peptides QAVTNPQNTLFAIK,SLEGDEPEPLPQVR and QGDLICLDGK as an example; these are shown in the plots below. As shown in panel A, we observe a large non-overlapping region in the middle of the temperature range. In order to approximate this area, we first calculate the distances between small molecule and control curve at temperatures where confidence intervals do not overlap. This is checked by conditional statements comparing the upper bounds and lower bounds of the respective confidence intervals. The distances can then be plotted against the temperature (panel B) and apex of the peak is found (X) to correctly classify the area as binding, aggregation or stabilisation. In the example, the area is classified as stabilisation. Everything that contributes to the approximated area is colored in red as shown in panel C.

However, there are some cases, where the approach needs slight adaptation. Given how the area is approximated, a stabilised peptide with increasing profile will lead to a negative stabilization score as shown in panels D and E, even though the peptide is stabilized by the small molecule. In order to account for these cases, we first need to determine the profile shape of individual peptides.

Another case that needs to be addressed are peptides, which show a non-monotonous curve shape and are stabilized by the small molecule. Often, the fitted curve upon addition of small molecule is then shifted towards higher temperatures as shown in panel G. This then leads to 2 areas, where one will have a positive sign and the other one will lead to a negative sum for the area (panel H). Again, we use the profile shapes to discriminate these cases. The stabilization score in such cases is then the absolute value of the largest area as shown in panel I. The sections below will go into more detail how this is achieved.

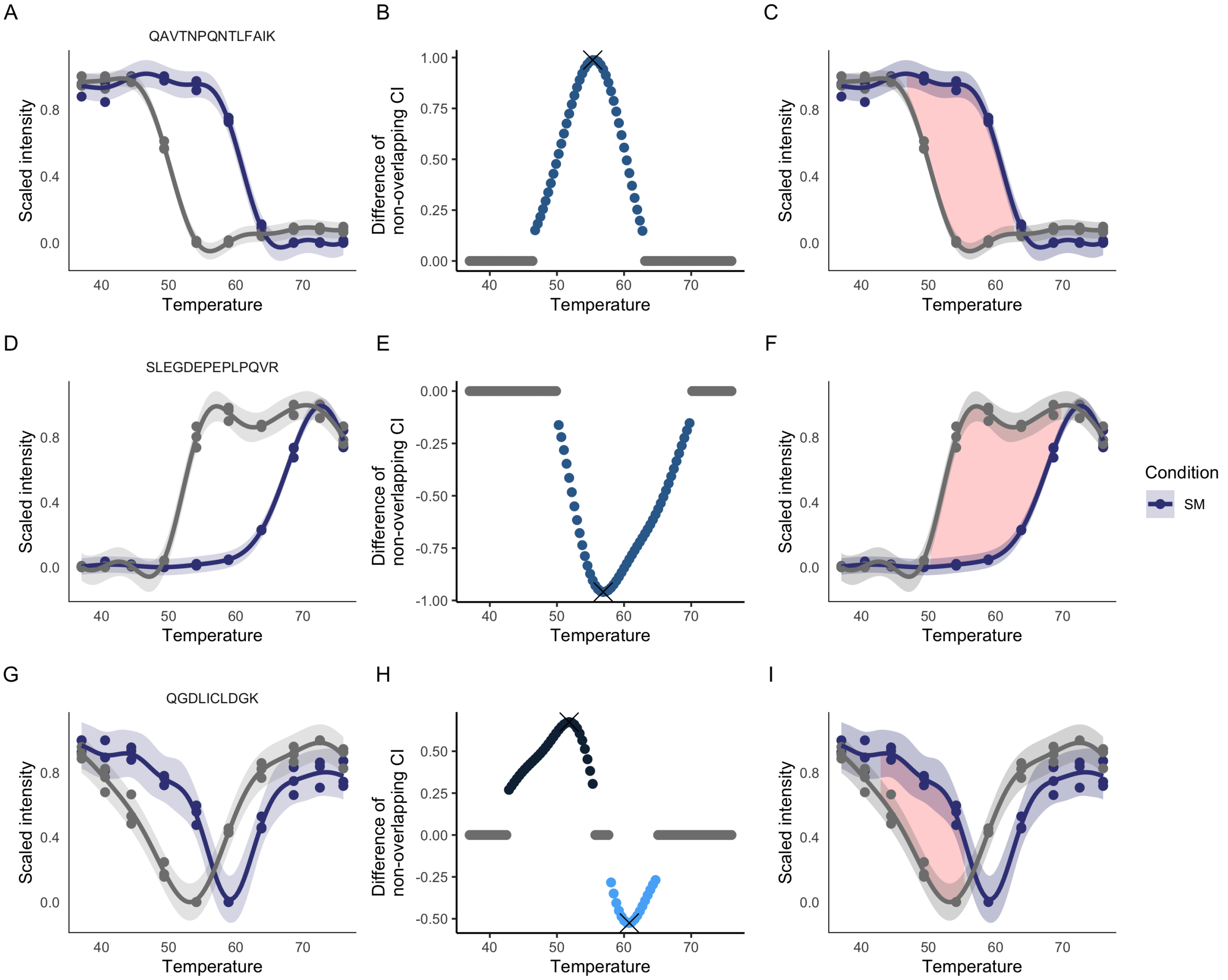

Example of how area is calculated for 3 indicated peptides. The first column shows the initial fit, second column the distance between the curves where confidence intervals do not overlap and the third column shows the peptide fits with highlighted area between the curves. The line represents the fit and the shaded area represents confidence intervals of the fit. The calculated area is highlighted in red.

6 Clustering

As highlighted above, we need to define the profile shape of each individual peptide in order to identify whether the area represents a stabilising or a destabilising effect. Without using the clustering output, stabilized peptides with increasing profile (see panel D above) will be wrongly classified as destabilization even thought the corresponding region is stabilised.

We observe four types of behaviour: Decreasing profile, Increasing profile, Non-monotonous profile (decreasing) and Non-monotonous profile (increasing). In the example above, the QAVTNPQNTLFAIK is classified as decreasing profile, while SLEGDEPEPLPQVR is classified as an increasing profile.
To classify all peptides into the different profile shapes, we first perform a fuzzy k-means clustering.

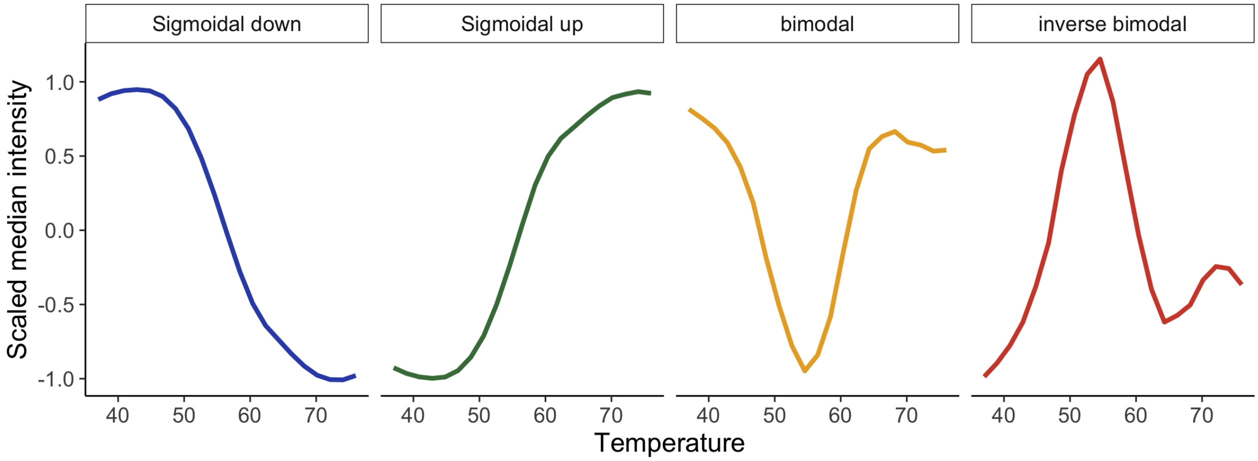

Median cluster profiles after clustering

Using the mean cluster shapes shown above, we define certain characteristics, based on which we then classify the curves into the different profiles shapes. The following characteristics based on the median profile at each temperature of each cluster are calculated:

- **intensity difference** = |IntensityT=37 - IntensityT=76|
- **maximal intensity difference** = (max(Intensity) + 2) - (min(Intensity) + 2)
- **monotonous curve shape** = |diff| < max(|diff|)/1.65

Median cluster profiles are then assigned to the previously defined profiles shapes using the flow chart shown below.

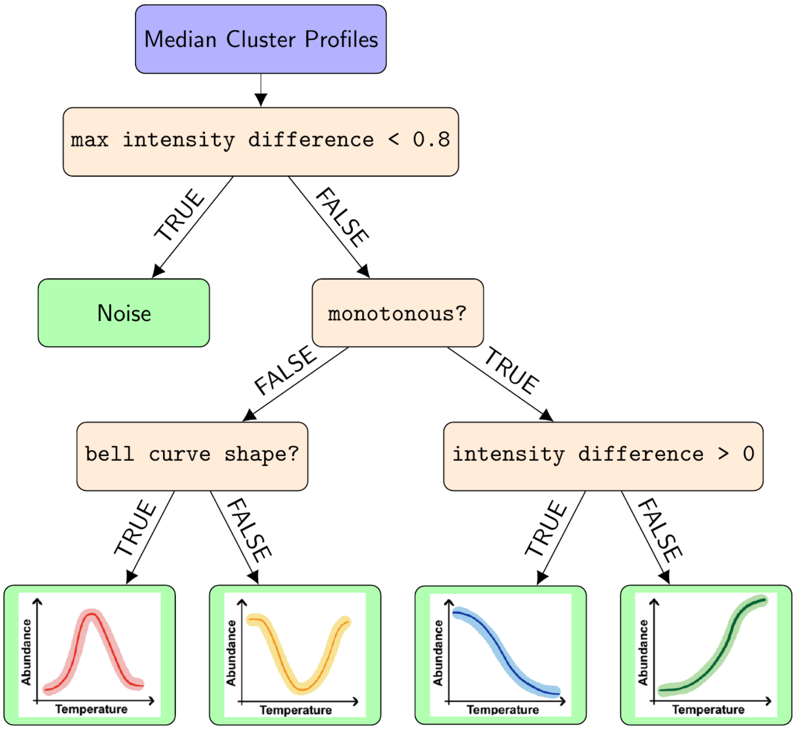

Clustering Overview

Going back to the examples of how area is calculated shown earlier, we then use the assigned clusters to determine the proper sign of the area. Given that SLEGDEPEPLPQVR has an increasing profile and its area initially has a negative sign due to how the area is calculated in the next step, this will be corrected by using the information of the profile shape and the score for this peptide is correctly classified as "stabilised".

7 Find significant peptides

As described above, we use clustering information as well as the calculated area between the curves to assign the 4 different effects (binding, (de-)stabilization and aggregation). Mainly, this function corrects the stabilization effect for peptides with an increasing profile for which initially destabilization would have been assigned due to the negative area. Furthermore, we correct for shifted non-monotonous curves being described as a change in aggregation instead of (de-)stabilization (as shown in panel I above).

8 Peptide fasta matching

Before aggregating scores from peptide level to protein level, we match peptides to their positions on the protein. Any peptide that will match to two different proteins or that still aligns to two different positions on the same protein will be discarded in this step.

9 Aggregate scores

Finally we calculate protein level stabilisation scores. To do so, we summarise the peptide level stabilisation scores into a protein level stabilisation score. This is achieved in 2 steps. First, we aggregate peptide-level scores of overlapping peptides to the amino acid level score. In the second step we aggregate amino-acid level score into a protein-level score. In both steps, we firstly calculate the mean of all scores, to determine whether the position/protein is overall stabilised (mean > 0) or destabilised (mean < 0). We then aggregate the score, using a 75% weighted quantile (weighted by the goodness of fit from the model) for stabilised positions and 25% weighted quantile for the destabilised positions. Next, we again use a 75% weighted quantile to aggregate the amino acid scores to a single protein level score for stabilised proteins and 25% weighted quantile for the destabilised proteins, shown in the two figures below.

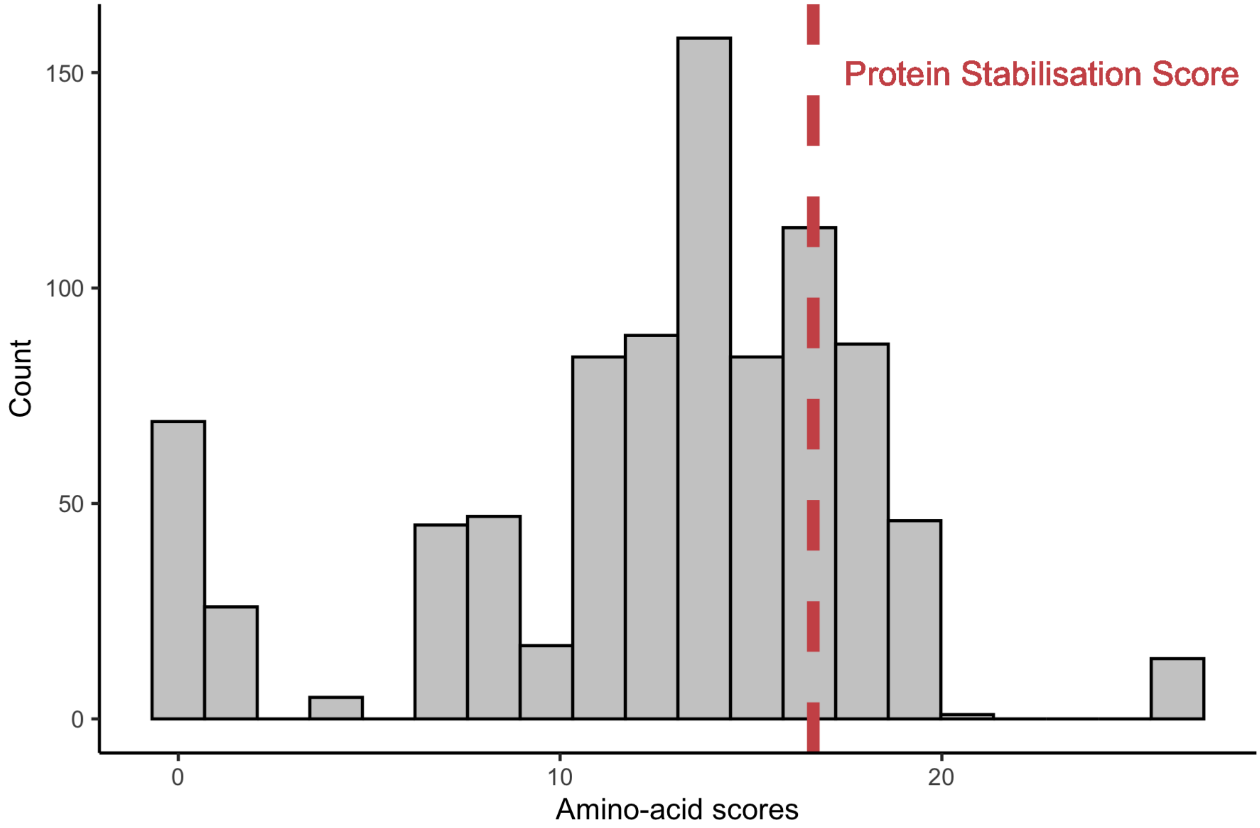

Example for aggregation of Amino-acid scores into protein-level stabilisation score. The histogram displays the distribution of Amino-acid scores for a specific protein. The red line indicates the 75% weighted quantile of the scores, which represents Protein-level stabilisation score.

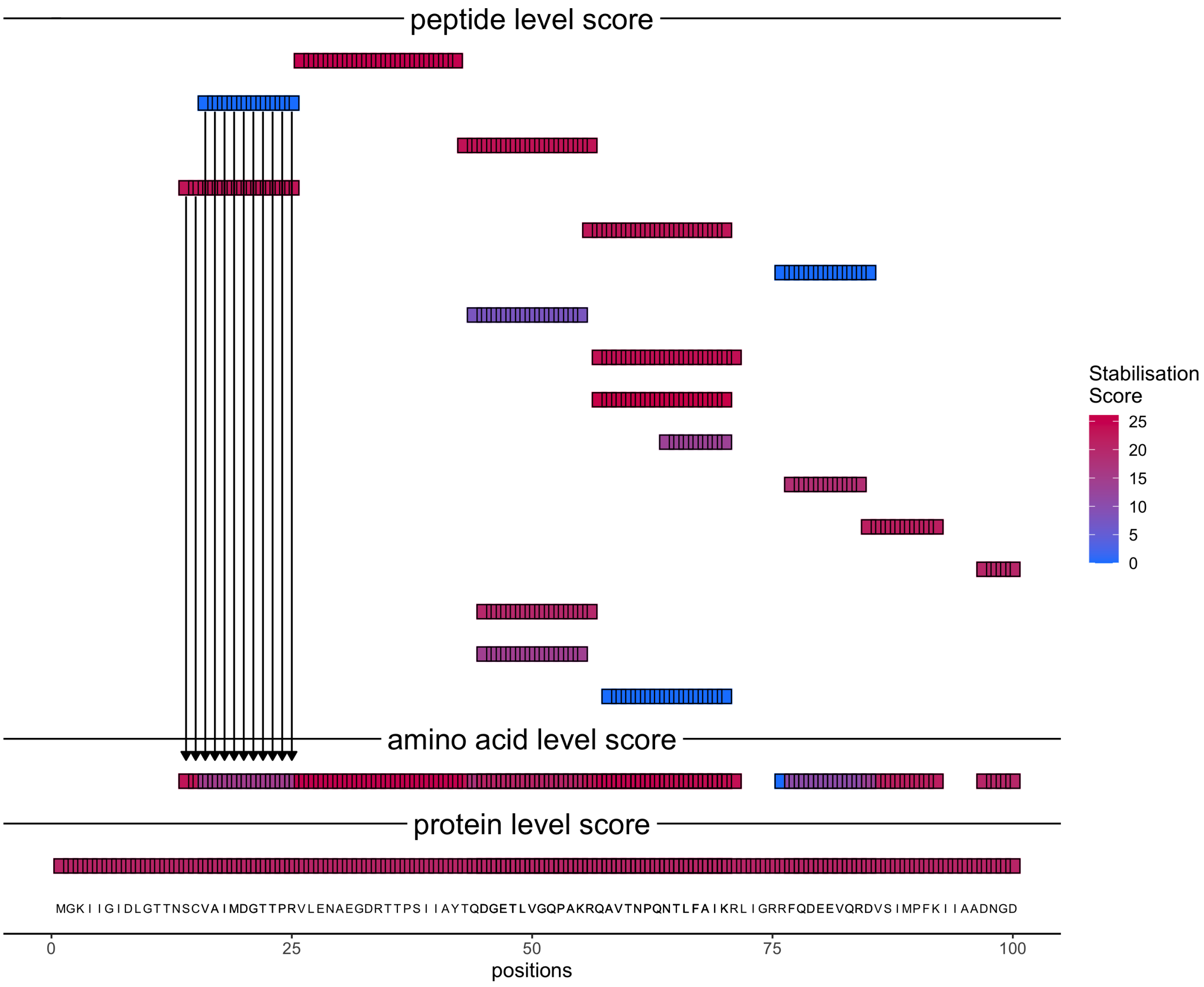

Illustrated example of score aggregation to protein level

Overall, the pipeline described above allows us to analyse LiP-MS based thermal profiling data. The pipeline firstly distinguishes between changes in binding, thermal stabilisation and aggregation. Next, the thermal stabilisation effect on peptide level is quantified. Last, peptide-level stabilisation score is summarised in protein-level stabilisation score. The analysis can be performed by running the pipeline as described below.

10 Running the pipeline

10.1 Setup

The analysis pipeline is dependent on the following R packages: data.table,dplyr,fclust,ggplot,ggpubr,magrittr,seqinr,spatstat,plo
With the following script, you can automatically check whether these dependencies are already installed.

```
source("scripts/install_R_dependencies.R")
check_dependencies()
```

Additionally, to run the GP fitting step, the following libraries have to be installed for python:

- scikit-learn==0.24.1
- matplotlib
- seaborn
- pandas

- jupyterlab
- pydot
- pillow
- tensorflow==2.4.1

Next, we need to specify some filepaths and load necessary functions to run the pipeline.

```
# load libraries
#### TO COMPLETE ####
# load functions
source("scripts/Spectronaut_to_python.R")
source("scripts/python_to_list.R",echo = FALSE)
source("scripts/area_calculation.R",echo = FALSE)
source("scripts/fuzzy_clustering.R",echo = FALSE)
source("scripts/post_analysis_functions.R",echo = FALSE)
source("scripts/find_significant_peptides.R",echo = FALSE)
source("scripts/peptide_fasta_matching.R",echo = FALSE)
source("scripts/aggregate_scores.R",echo = FALSE)

# specify path to python distribution
use_python("PATH/TO/PYTHON/DISTRIBUTION")
source_python("scripts/fitGP_function_sm.py")

# specify where output will be saved
out_dir <- "FILEPATH"

# name of small molecule
small_molecule <- "ATP"

# Filepath of spectronaut export
spectronaut_file_path <- "FILEPATH/Spectronaut_export.tsv"

# Filepath to annotation file
annotation_file <- "FILEPATH/Annotation_table.csv"
```

10.2 Spectronaut_to_python()

```
# read spectronaut export
spectronaut_data <- read.delim(spectronaut_file_path,header = T)

Spectronaut_to_python(small_molecule,spectronaut_data,annotation_file,out_dir)

# creates the following directories with files:
# - OUTPUT_FILEPATH/ATP/python_import/ATP_FT.csv
# - OUTPUT_FILEPATH/ATP/python_import/ATP_HT.csv
```

10.3 GP_fitting()

```
# generate file paths
file_path_FT <- paste0(out_dir,"/",small_molecule,"/","python_import/",small_molecule,"_FT.csv")
file_path_HT <- paste0(out_dir,"/",small_molecule,"/","python_import/",small_molecule,"_HT.csv")
```

```
# fully tryptic peptides
FitGPs_function(r.small_molecule,r.file_path_FT,"FT",r.out_dir)

# half tryptic peptides
```

```
FitGPs_function(r.small_molecule,r.file_path_HT,"HT",r.out_dir)

# creates the following directories and files:
# - OUTPUT_FILEPATH/ATP/python_output/MLL_ATP_FT.csv
# - OUTPUT_FILEPATH/ATP/python_import/solution_ATP_FT.csv
# - OUTPUT_FILEPATH/ATP/python_output/MLL_ATP_HT.csv
# - OUTPUT_FILEPATH/ATP/python_import/solution_ATP_HT.csv
```

10.4 Python_to_list()

```
all_results <- python_to_list(small_molecule,spectronaut_data,out_dir)
```

10.5 Calculate_area()

```
calculated_area <- area_calculation_2(all_results,small_molecule,cutoff = 0.5,
                                      control = "Control")
calculated_area <- assign_peak_apex(calculated_area)
calculated_area$define_peak <- ifelse(calculated_area$define_new_peak == "not_aggregation",
                                      "stabilisation",calculated_area$define_peak)
```

10.6 Clustering()

```
clusters <- fuzzy_clusters_two_conditions(all_results,small_molecule,control = "Control", k = 15,
                                          show_clusters = TRUE)
```

10.7 Find_significant_peptides()

```
significant_all <- find_significant_peptides(calculated_area,clusters,
                                             small_molecule,control = "Control")
```

10.8 Peptide_fasta_matching()

```
fasta_ecoli <- read.fasta("~/polybox/SemPro_Picotti/001_ATP/databases/211129_Ecoli_proteome_fasta.fasta
all_peptides <- significant_all$scores$Peptide %>% unlist() %>% unique()

matched_peptides <- peptide_fasta_matching(all_peptides,fasta_ecoli)
```

10.9 Aggregate_scores()

```
aggregated_scores <- score_aggregation(significant_all,small_molecule,
                                       matched_peptides)
```
