# Supplementary material for: In situ analysis of osmolyte mechanisms of proteome thermal stabilization
Source: Nat Chem Biol. 2024 Feb 29;20(8):1053–65. doi: 10.1038/s41589-024-01568-7 (PMC11288892; doi:10.1038/s41589-024-01568-7)
Supplement: Supplementary file 2 — Reporting Summary [file 41589_2024_1568_MOESM2_ESM.pdf]

Corresponding author(s): Paola Picotti

Last updated by author(s): 01/04/2024

Reporting Summary

Nature Portfolio wishes to improve the reproducibility of the work that we publish. This form provides structure for consistency and transparency in reporting. For further information on Nature Portfolio policies, see our Editorial Policies and the Editorial Policy Checklist.

Statistics

For all statistical analyses, confirm that the following items are present in the figure legend, table legend, main text, or Methods section.

n/a	Confirmed	
☐	☒	The exact sample size (*n*) for each experimental group/condition, given as a discrete number and unit of measurement
☒	☐	A statement on whether measurements were taken from distinct samples or whether the same sample was measured repeatedly
☐	☒	The statistical test(s) used AND whether they are one- or two-sided<br>*Only common tests should be described solely by name; describe more complex techniques in the Methods section.*
☒	☐	A description of all covariates tested
☐	☒	A description of any assumptions or corrections, such as tests of normality and adjustment for multiple comparisons
☐	☒	A full description of the statistical parameters including central tendency (e.g. means) or other basic estimates (e.g. regression coefficient) AND variation (e.g. standard deviation) or associated estimates of uncertainty (e.g. confidence intervals)
☐	☒	For null hypothesis testing, the test statistic (e.g. *F*, *t*, *r*) with confidence intervals, effect sizes, degrees of freedom and *P* value noted<br>*Give P values as exact values whenever suitable.*
☒	☐	For Bayesian analysis, information on the choice of priors and Markov chain Monte Carlo settings
☒	☐	For hierarchical and complex designs, identification of the appropriate level for tests and full reporting of outcomes
☐	☒	Estimates of effect sizes (e.g. Cohen's *d*, Pearson's *r*), indicating how they were calculated

Our web collection on statistics for biologists contains articles on many of the points above.

Software and code

Policy information about availability of computer code

Data collection	Xcalibur 4.2
Data analysis	Spectronaut (14.5, 14.11), Spectromine (2.3.200924.47784), R (4.0.5), Python (3.9.4), gpytorch (1.4.2), topGO (2.40.0), Tango (2.2), pymol (2.4.0), CamSol, Aggrescan. The original code used for analysis is deposited on Github https://github.com/PicottiGroup/Thermal_unfolding

For manuscripts utilizing custom algorithms or software that are central to the research but not yet described in published literature, software must be made available to editors and reviewers. We strongly encourage code deposition in a community repository (e.g. GitHub). See the Nature Portfolio guidelines for submitting code & software for further information.

Data

Policy information about availability of data

All manuscripts must include a data availability statement. This statement should provide the following information, where applicable:

- Accession codes, unique identifiers, or web links for publicly available datasets
- A description of any restrictions on data availability
- For clinical datasets or third party data, please ensure that the statement adheres to our policy

All mass spectrometry proteomics data have been deposited at ProteomeXchange Consortium via the PRIDE partner repository with the dataset identifier: PXD036186 (Username: reviewer_pxd036186@ebi.ac.uk and Password: XBU0XEH4). UniProt fasta databases for E. coli (strain K12, organism ID 83333) was accessed in November 2020 via the UniProt databases download page (https://www.uniprot.org/downloads). Protein structures for DnaK (PDBID: 4JNE), was

downloaded in 2022 from the Protein Data Bank website (https://www.rcsb.org/pdb). AlphaFold predictions for the whole E. coli proteome were downloaded in August 2021. DescribeProt database for the whole E. coli proteome (http://biomine.cs.vcu.edu/servers/DESCRIBEPROT/download.html) was downloaded in February 2021. The TPP dataset from published study (https://doi.org/10.15252/msb.20188242, Dataset EV3 of the publication) was downloaded in 2020. All other data needed to evaluate the conclusions in the paper are present in the supplementary materials.

Human research participants

Policy information about studies involving human research participants and Sex and Gender in Research.

Reporting on sex and gender	Does not apply. No human research participants.
Population characteristics	Does not apply.
Recruitment	Does not apply.
Ethics oversight	Does not apply.

Note that full information on the approval of the study protocol must also be provided in the manuscript.

Field-specific reporting

Please select the one below that is the best fit for your research. If you are not sure, read the appropriate sections before making your selection.

☒ Life sciences ☐ Behavioural & social sciences ☐ Ecological, evolutionary & environmental sciences

For a reference copy of the document with all sections, see nature.com/documents/nr-reporting-summary-flat.pdf

Life sciences study design

All studies must disclose on these points even when the disclosure is negative.

Sample size	The sample size (10 temperatures per condition) were selected because of the technical limitation - the number of chambers in the PCR instrument. The two replicates per temperature were selected to increase the statistical power and at the same time considering the measurement time and feasibility of a large experiment. We also tested the effects of 6 osmolytes, which represent all major classes of these molecules.
Data exclusions	No replicates/measured samples were excluded from the analysis. Within a measured dataset, peptides with more than 20% of missing values were excluded from fitting. After the GP modeling, the data was filtered based on the goodness of fit. This was performed in order to avoid the bias for highly abundant proteins with higher quality of data and better reproducibility. Similar filtering approach was established in the preceeding study (Leuenberger et al, Science 2017)
Replication	Thermal profiling of lysates were done in duplicate for each condition. Our major conclusions were replicated between E. coli and human. DSF and other biochemical assays were done in triplicate or quadruplicate.
Randomization	Osmolytes were randomly distributed in batches. The batch size was designed in a way that it was feasible to perform the experiment in a single day. Experiment for control condition was performed in each batch and the individual osmolyte condition was always compared only to the control condition from the same batch. Within the batch, samples were grouped in a set of 10 (the temperature gradient). Within the group of 10, samples could not be randomised because of the technical limitations of experimental setup. However, the groups of 10 samples within a batch were randomised both in the experimental setup (LiP-MS experiment) and the running order for the MS acquisition.
Blinding	Does not apply. Our study was not designed to make comparisons between groups. Our experiments were conducted on all mass spectrometrically detected proteins in each lysate under study.

Reporting for specific materials, systems and methods

We require information from authors about some types of materials, experimental systems and methods used in many studies. Here, indicate whether each material, system or method listed is relevant to your study. If you are not sure if a list item applies to your research, read the appropriate section before selecting a response.

Materials & experimental systems

n/a	Involved in the study
☒	☐ Antibodies
☐	☒ Eukaryotic cell lines
☒	☐ Palaeontology and archaeology
☒	☐ Animals and other organisms
☒	☐ Clinical data
☒	☐ Dual use research of concern

Methods

n/a	Involved in the study
☒	☐ ChIP-seq
☒	☐ Flow cytometry
☒	☐ MRI-based neuroimaging

Eukaryotic cell lines

Policy information about cell lines and Sex and Gender in Research

Cell line source(s)	We used a lab stock of HEK293 cells.
Authentication	We did not authenticate the cell line. The cell lysate was used purely as a source of the human proteome.
Mycoplasma contamination	We did not test the cell line for Mycoplasma contamination.
Commonly misidentified lines (See ICLAC register)	None.
